# Abortion in Zimbabwe: A national study of the incidence of induced abortion, unintended pregnancy and post-abortion care in 2016

**DOI:** 10.1371/journal.pone.0205239

**Published:** 2018-10-24

**Authors:** Elizabeth A. Sully, Mugove Gerald Madziyire, Taylor Riley, Ann M. Moore, Marjorie Crowell, Margaret Tambudzai Nyandoro, Bernard Madzima, Tsungai Chipato

**Affiliations:** 1 Guttmacher Institute, New York, New York, United States of America; 2 University of Zimbabwe College of Health Science–Clinical Trials Unit (UZCHS-CTU), Harare, Zimbabwe; 3 Zimbabwe Ministry of Health and Child Care, Harare, Zimbabwe; Instituto Nacional de Salud Publica, MEXICO

## Abstract

**Background:**

Zimbabwe has the highest contraceptive prevalence rate in sub-Saharan Africa, but also one of the highest maternal mortality ratios in the world. Little is known, however, about the incidence of abortion and post-abortion care (PAC) in Zimbabwe. Access to legal abortion is rare, and limited to circumstances of rape, incest, fetal impairment, or to save the woman’s life.

**Objectives:**

This paper estimates a) the national provision of PAC, b) the first-ever national incidence of induced abortion in Zimbabwe, and c) the proportion of pregnancies that are unintended.

**Methods:**

We use the Abortion Incidence Complications Method (AICM), which indirectly estimates the incidence of induced abortion by obtaining a national estimate of PAC cases, and then estimates what proportion of all induced abortions in the country would result in women receiving PAC. Three national surveys were conducted in 2016: a census of health facilities with PAC capacity (n = 227), a prospective survey of women seeking abortion-related care in a nationally-representative sample of those facilities (n = 127 facilities), and a purposive sample of experts knowledgeable about abortion in Zimbabwe (n = 118). The estimate of induced abortion, along with census and Demographic Health Survey data was used to estimate unintended pregnancy.

**Results:**

There were an estimated 25,245 PAC patients treated in Zimbabwe in 2016, but there were critical gaps in their care, including stock-outs of essential PAC medicines at half of facilities. Approximately 66,847 induced abortions (uncertainty interval (UI): 54,000–86,171) occurred in Zimbabwe in 2016, which translates to a national rate of 17.8 (UI: 14.4–22.9) abortions per 1,000 women 15–49. Overall, 40% of pregnancies were unintended in 2016, and one-quarter of all unintended pregnancies ended in abortion.

**Conclusion:**

Zimbabwe has one of the lowest abortion rates in sub-Saharan Africa, likely due to high rates of contraceptive use. There are gaps in the health care system affecting the provision of quality PAC, potentially due to the prolonged economic crisis. These findings can inform and improve policies and programs addressing unsafe abortion and PAC in Zimbabwe.

## Introduction

Over the past two decades, Zimbabwe has undergone a period of extended economic crisis and declining standards of living [1]. Despite these challenges, it has maintained a strong family planning program with one of the highest contraceptive prevalence rates in sub-Saharan Africa; 48% of all women and 66% of currently married women in Zimbabwe are using a modern contraceptive method [2]. However, high unmet need persists among unmarried and adolescent women [2]. These gaps in Zimbabwe’s family planning program leave women at risk of experiencing an unintended pregnancy and abortion, but very little is known about the incidence of abortion or unintended pregnancy in the country.

Zimbabwe has a restrictive abortion law, with legal abortion limited to circumstances of rape, incest, fetal impairment, or to save the woman’s life [3]. In practice, however, access to legal abortions on these grounds is difficult and rare [4]. Legal and administrative barriers coupled with stigma among both women and providers [5] and fear of social repercussions [6] serve to limit access to legal abortion in Zimbabwe. Rather than reduce abortion incidence, restrictive abortion laws like that of Zimbabwe result in women pursuing clandestine and potentially unsafe abortions [7].

The safety of abortion is of particular concern in Zimbabwe: the country has one of the highest maternal mortality ratios in the world, estimated at 651 per 100,000 live births [2]. In contrast to the global trend of reductions in maternal mortality over the past 25 years, which was facilitated by countries striving to meet Millennium Development Goal 5, Zimbabwe has experienced an increase in its maternal mortality ratio [8]. Unsafe abortion is likely a contributing cause of maternal mortality in Zimbabwe. An estimated 25 million unsafe abortions took place globally each year between 2010 and 2014 [7] and abortion (including miscarriages and ectopic pregnancies) accounted for 9% of maternal deaths in 2016 [9]. However, little is known about unsafe abortion in Zimbabwe. The most recent source of data on the contribution of abortion to maternal mortality is from 2007; at that time, abortion complications were one of the top five causes of maternal mortality, accounting for 5.8% of maternal deaths [10]. However, it is extremely difficult to accurately capture maternal deaths associated with unsafe abortion, especially in contexts where abortion is highly stigmatized.

The Zimbabwe Ministry of Health and Child Care (MoHCC) has made efforts to reduce maternal mortality through improving post-abortion care (PAC) services; this includes launching training programs on manual vacuum aspiration (MVA) in 2008 [6,11], including misoprostol on the Essential Drugs List in 2011, piloting training on using misoprostol for PAC in 2013 [11], and revising The National Guidelines for Comprehensive Abortion Care in Zimbabwe in 2014 to expand PAC to primary care facilities and ensure PAC services are free in public facilities [12].

There are no national estimates of the incidence of abortion or the national provision of PAC in Zimbabwe. This study provides the first nationally representative estimate of the provision of PAC, and the incidence of induced abortion and unintended pregnancy in Zimbabwe. These findings have the potential to inform policy and programs addressing unsafe abortion and PAC in Zimbabwe.

## Data and methods

### Overview

This paper uses the Abortion Incidence Complications Method (AICM) [13] to estimate the incidence of induced abortion in Zimbabwe. The AICM has been widely applied in over 25 countries worldwide, including 10 in sub-Saharan Africa [14–27]. The methodology indirectly estimates the incidence of abortion by obtaining a national estimate of the number of PAC cases treated in facilities, and then estimating what proportion of all abortions in the country would result in women receiving PAC.

The AICM includes a Health Facilities Survey (HFS) consisting of interviews with PAC service providers and a Health Professionals Survey (HPS) with purposively-selected key informants who are knowledgeable about abortion. This study also includes a Prospective Morbidity Survey (PMS), which is a nationally representative, facility-based study that collects prospective data from PAC patients to measure PAC service provision at the facility-level. These three surveys were conducted from August to November 2016. Ethical approval was obtained from the Medical Research Council of Zimbabwe, the Joint Research Ethics Committee for the University of Zimbabwe, College of Health Sciences, and the Parirenyatwa Group of Hospitals and the Guttmacher Institute’s Institutional Review Board.

### Data

#### Health Facilities Survey (HFS)

The HFS gathers retrospective data on the number of PAC cases treated annually. All public, private and non-governmental organization (NGO) health facilities that have the capacity to provide PAC were eligible to participate in the survey. Health facilities in Zimbabwe are capable of providing PAC if they have an operating theatre or if staff have been trained to use misoprostol for PAC [12]. We obtained lists of all known health facilities from the MoHCC, the Health Professionals Authority (where all public and private facilities are required to register), the Private Hospitals Association of Zimbabwe, the Association of Health Funders of Zimbabwe, and Population Services-Zimbabwe, a local NGO. We excluded specialized facilities unrelated to PAC, as well as duplicate facilities, individual doctors, and facilities that lacked the capacity to provide PAC.

According to the National Guidelines for Comprehensive Abortion Care in Zimbabwe, the lowest level of public health facilities (primary health centers) can only provide PAC using misoprostol if they have trained providers [12]. Only 63 primary health centers meet these criteria, having been included in a 2013 operations research study conducted by MoHCC and Venture Strategies Innovations [11].

Based on the inclusion criteria, we identified 245 facilities in Zimbabwe with the capacity to provide PAC (Table 1). During fielding, 7% of facilities on the list were not eligible for the survey. There was a 99.6% response rate in all eligible facilities. Overall, 227 facilities participated in the HFS (Table 1).

**Table 1:** Universe and sample selection, by type of facility and survey, Zimbabwe 2016.

	HFS Census ^a^		PMS Sample ^b^		MoHCC M&E ^d^
**Type of health facility**	Response rate (%)	Facilities interviewed (No.)	Sample proportion from universe of facilities with PAC capacity (%)	Facilities interviewed (No.) ^c^	Number of facilities
Primary health centers	100%	59	30%	18	822
District and mission hospitals	100%	89	52%	47	3
Provincial hospitals	100%	8	100%	8	
Central hospitals	100%	5	100%	5	
Private hospitals	97%	32	77%	26	
NGO	100%	34	68%	23	
**Total**	99.6%	227	56%	127	825

a Eligible is defined as a facility having the capacity to provide post-abortion care (PAC). Seven percent of facilities in the HFS were not eligible, this included duplicates (n = 3), closed or reclassified facilities (n = 6), and facilities without PAC capacity or not providing PAC (n = 8).

b Facilities were added to the PMS to account for misclassification by province. We included additional facilities to have 100% of facilities in that province at the level where misclassification occurred. This included one private hospital, and six NGO facilities. Seven percent of facilities in the PMS were not eligible.

c For the PMS, primary health centers, district and mission hospitals, and provincial hospitals had a 100% response rate. The lowest response rate was among NGO facilities at 82% responding, and private hospitals had a 96% response rate.

d Ministry of Health and Child Care (MoHCC) monitoring and evaluation (M&E) data includes public facilities that do not have PAC capacity and therefore were not in the sampled universe. However, they still get PAC patients and report these numbers to the MoHCC, so we included out-of-sample MoHCC facilities to calculate PAC caseloads in Zimbabwe.

One respondent who had worked at the facility for at least six months was selected at each participating facility. HFS respondents were selected based on their knowledge of the facility’s PAC services; most often, this was a nurse/midwife (68%), senior nurse (27%), or doctor/hospital administrator (5%). Written informed consent and face-to-face structured questionnaires were administered to HFS respondents by Provincial Reproductive Health Officers (RHOs), who are trained health professionals working for the MoHCC. Information was collected on PAC caseloads, facility infrastructure, functionality and availability of equipment and medicines, and PAC procedures used. (S1 File).

#### Prospective Morbidity Survey (PMS)

The PMS was conducted in a nationally representative sample of facilities with PAC capacity, using the HFS universe as the sampling frame. Facilities were stratified by level, ownership and province. The PMS sample included 56% of facilities in Zimbabwe with PAC capacity: all central hospitals (n = 5), provincial hospitals (n = 8), and higher-level private facilities (n = 19) were included due to their anticipated large caseloads; sampling proportions were smaller for lower-level facilities (Table 1). Overall, 127 facilities took part in the PMS, which corresponds to a facility response rate of 95% (Table 1).

At the 127 participating facilities, data was collected on all women presenting at these facilities for PAC during the 28-day study period. Questionnaires administered to both patients and their providers were used to capture a count of the number of women who came in for PAC (S2 File). Verbal informed consent was obtained from the woman for both her interview, as well as for the interview with her provider. Interviewers provided and read to women the consent form, after which interviewers indicated on the form whether consent was obtained, and verified this by providing their own signature, name and date. Parental or guardian consent was not obtained for minors as pregnant (or previously pregnant) minors are considered emancipated in Zimbabwe. The three ethics committees approved this informed consent procedure.

Facility-level aggregate information, rather than individual-level responses, were used to calculate PAC caseload totals for this analysis. A number of steps were taken to minimize missed PAC patients from the PMS caseload total. Interviewers were given tracking forms to complete each day where they recorded the number of completed interviews, the number of women with post-abortion complications so severe that they were unable to consent to participate in the survey, the number of maternal deaths, and the number of cases they missed. Interviewers were trained to consult with colleagues across multiple departments within the facilities in order to keep track of any PAC cases presenting in the facility at any time over the 28-day period.

#### Health Professionals Survey (HPS)

The HPS collected information from experts on abortion, abortion-related complications, and access to PAC in Zimbabwe. The study team developed a purposive list of 125 key informants, covering all provinces, which included doctors, nurses, midwives, policy makers, lawyers, pharmacists, traditional health workers, village chiefs, NGO staff, and other individuals well placed to know about the behaviors, outcomes and treatment of women seeking abortions. An effort was made to have sufficient representation of experts with knowledge of rural areas. Three-quarters of the sample were health providers, and the remaining quarter were selected for their knowledge of women’s health issues at the community level. Respondents to the HPS were on average 42 years old, with 14 years of work experience and 60% were female.

Six HPS respondents were excluded from the final dataset as they had been incorrectly selected for the survey. These were respondents who struggled to answer the questions, and were assessed as having low knowledge on the topic by both the interviewer and themselves. Only one selected respondent refused to participate in the survey. The final sample size for the HPS was 118.

Through face-to-face structured interviews conducted by four highly skilled professionals, respondents were asked about from whom women seek abortions, the likelihood of women experiencing complications, the proportion of women with complications that would receive treatment and about Zimbabwean women seeking abortions outside of the country (S3 File). We designed a visual aid to facilitate data collection for the HPS, due to the complex nature and nesting of questions (S4 File). Given that respondents may not have had enough knowledge to answer all the questions, we also asked respondents how certain they were (on a scale of one to ten) of their answers for abortion in urban and rural areas, and for misoprostol use in urban and rural areas (a more recently introduced abortion method in the country).

#### Other data sources

To calculate abortions rates, we used data on the population of reproductive age women in 2016, nationally and by province, from the Zimbabwe National Statistics Agency Population Projections Report based on the 2012 National Census [28]. We calculated the national and provincial number of births, from age-specific fertility rates from the 2015 Zimbabwe Demographic and Health Survey (ZDHS) [2] applied to the female population by age from the Zimbabwe National Statistics Agency (ZNSA) [28]. To determine the distribution of women based on wealth and place of residence, the Poverty and Poverty Datum Line Analysis Report from 2012 produced by the ZNSA provided the wealth distribution [29] and the ZDHS 2015 provided the urban and rural distribution [2]. The ZDHS was used to estimate unintended and intended births to calculate the total number of unintended pregnancies [2].

### Terminology

The HFS and PMS surveys were designed to measure the number of women arriving at a facility seeking PAC due to complications from both miscarriages and induced abortions. The HPS measures the likelihood that women having complications from induced abortions would receive treatment. Abortion complications were defined in all three surveys as not only the extremely serious cases such as those with sepsis or a perforated uterus, but also incomplete abortions, which are usually identified by heavy bleeding and present a somewhat less severe health risk to the woman but still requires treatment in a health facility. Respondents were instructed that abortion complications do not include the expected bleeding associated with a miscarriage or induced abortion that would resolve itself on its own, and does not require treatment. However, in a clinical setting some of these cases may be indistinguishable; incomplete abortions that would resolve on their own may still receive PAC alongside incomplete abortions that do require treatment to be resolved.

There is likely measurement error in respondent’s assessment of abortion complications, and PAC cases enumerated by respondents probably include some induced abortions and miscarriages that did not in fact require treatment but nonetheless received it. We therefore refer to estimates from the HFS and PMS as PAC cases, to acknowledge that not all women receiving PAC have abortion complications.

### Steps in estimating abortion incidence

**Fig 1:**
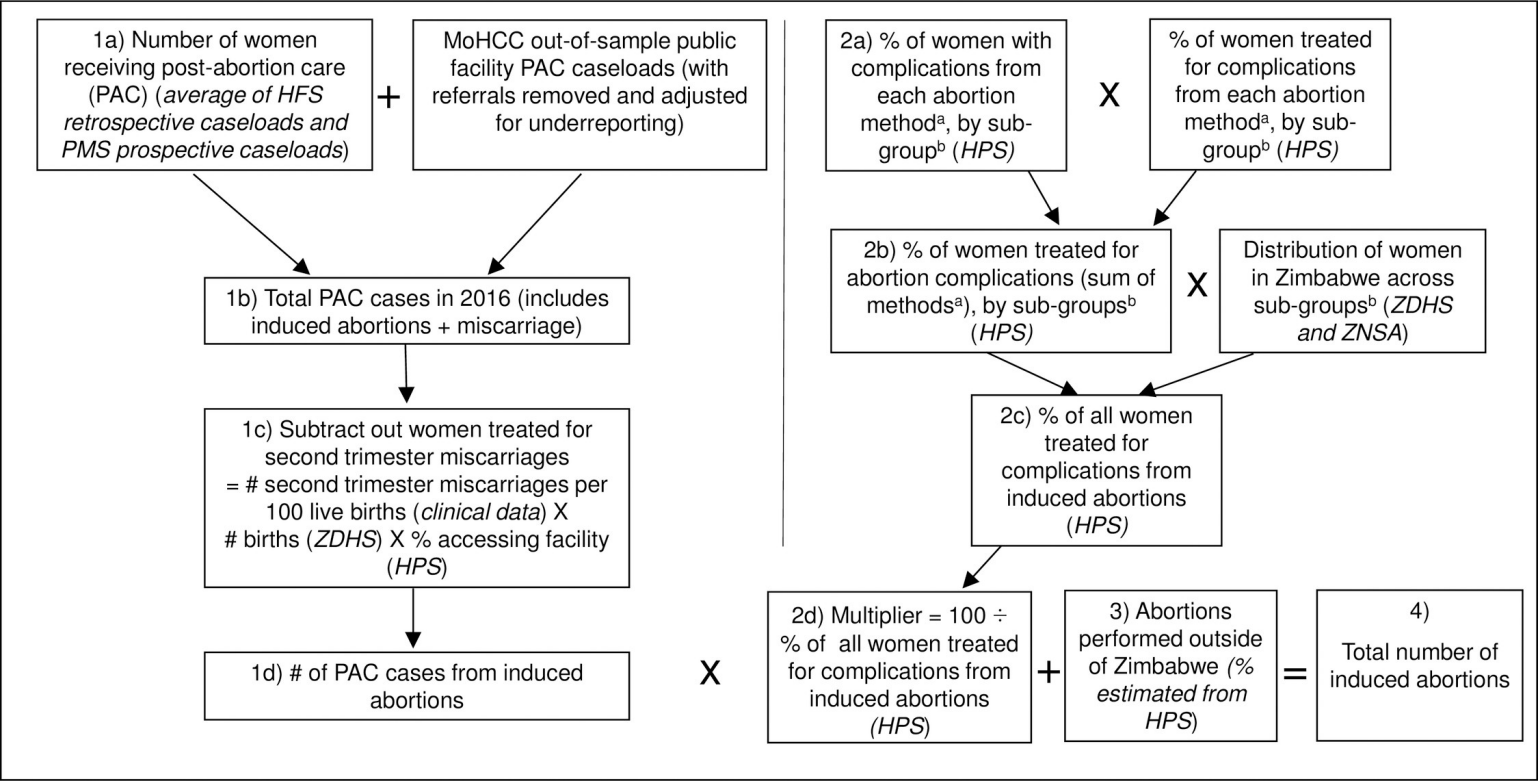
Abortion Incidence Complications Methodology (AICM), with adjustments made for Zimbabwe. ^a^ Abortion methods include surgical methods, misoprostol, and other methods of abortion. ^b^ Sub-groups include rural poor women, rural non-poor women, urban poor women, urban non-poor women.

The following details each step of the AICM. Mathematical Appendix A in S1 Appendix provides the detailed equations for the AICM calculations, and Fig 1 diagrams the methodology’s key components and data sources. We calculated abortion incidence nationally and by geographic region. We did not calculate provincial-level estimates as women do not always receive treatment in the provinces in which they reside. Instead, we constructed three geographical regions that included the provinces with higher-level facilities that are likely to treat women from the surrounding provinces. These three regions were Matabeleland (Bulawayo, Matabeleland North, Matabeleland South, Midlands), Mashonaland (Harare, Mashonaland East, Mashonaland West, Mashonaland Central) and South Eastern Region (Manicaland and Masvingo). All analyses were conducted in Stata 14.2 and weighted for non-response.

#### (1) Number of women receiving post-abortion care

The HFS and PMS obtained estimates of PAC patients (inpatient and outpatient) treated for either miscarriage or induced abortion. HFS respondents were asked about PAC cases in the past month and a typical month to account for possible seasonality of obstetric care and minimize reporting errors. Respondents who had difficulty reporting in the month time frame (this usually happened when the caseloads were very small) could provide the information for a typical year and the past year. PAC cases were estimated in the PMS using prospective estimates of PAC patients from both completed interviews and missed cases captured on the tracking sheets. Both PMS and HFS estimates were adjusted for referrals to avoid double-counting of PAC patients who may have been treated at one facility and then referred for additional care to a higher level facility (S2 Appendix). In addition to measuring PAC caseloads at each facility, we also assess the capacity of facilities to provide PAC, including the availability of essential drugs, tests and products, and the most commonly used procedures for PAC.

The annual number of women receiving PAC at a facility was calculated as the average of all available data points from our study. For facilities in the PMS, this included the past month estimate, the typical month estimate, and prospectively measured caseloads. For all other facilities, only the past month and typical month estimates were used. Facility-level PAC estimates were then weighted to account for non-response in the HFS to provide national and regional estimates of the number of women who obtained PAC in 2016. Confidence intervals are not applicable for the facility-level PAC caseload estimates as the HFS was conducted as a census, rather than a sample.

In the process of validating the assumption to exclude official health statistics due to underreporting [30], we uncovered PAC cases in the MoHCC data being reported from facilities outside of our sample. These were public primary health centers that had not received training to use misoprostol as well as three district hospitals that had been erroneously missed from the HFS facility listing (n = 825, Table 1). The HFS data collectors conducted a brief phone survey with a sample of these primary health centers and all three district hospitals to assess the accuracy of these reported PAC cases (S3 Appendix). While most cases in the out-of-sample public facilities were referred to higher-level facilities due to the lack of PAC capacity, we determined that out-of-sample facilities were providing some, though often insufficient, PAC treatment. We therefore took the reported MoHCC caseloads and subtracted the reported proportion referred. We also applied an underreporting ratio since the referral-adjusted out-of-sample caseloads are likely an underestimate (S3 Appendix). This total out-of-sample caseload was added to the total caseload estimate from the HFS and PMS to estimate the total annual PAC caseload in Zimbabwe.

#### (2) Adjustment for PAC cases due to miscarriage

PAC cases include women seeking care for both induced abortions and miscarriages. The second step of the AICM is estimating the proportion of all PAC cases that are due to induced abortions. To do this we make two key assumptions. The first assumption is that women would likely only seek PAC for miscarriages in the second trimester (13–22 weeks gestation), as miscarriages in the first trimester would resolve on their own. The second assumption is that not all women who need treatment would be able to receive it due to access, costs, knowledge, or a variety of other reasons, including death.

To quantify the number of women having miscarriages in the second trimester, we estimated the number of miscarriages using clinical data on the risk of miscarriage by gestational age in the US, as these are the only data which exist [31]. We used life table estimates provided by Harlap et al. to calculate a risk of miscarriage between 13–22 weeks, which is 3.41 miscarriages per 100 live births [31]. To quantify the number of women having second trimester miscarriages who would receive care, we used two sources of data. Previous AICM studies have assumed that the treatment-seeking rate for a second trimester miscarriage is equivalent to the proportion of women delivering in a health facility. Delivering in a health facility is seen as the nearest measure of women’s access to obstetric and gynecological care in the absence of specific data on access to PAC services. We also asked HPS respondents what proportion of women with second trimester miscarriages would receive PAC. Those two sources of facility access were within 3 percentage points, so we used the PAC-specific estimate from the HPS. With these two assumptions, we subtracted the second trimester miscarriage cases receiving treatment from the PAC caseload total to arrive at the number of PAC cases due to induced abortion.

#### (3) Estimating the multiplier

The HPS was used to calculate a multiplier, or an estimate of the number of women having abortions who either did not experience a complication or did have a complication but did not receive treatment, for every one woman receiving PAC. As complications and treatment access are likely to vary based on where a woman lives and what resources she has, we asked about complications and treatment estimates separately for four sub-groups of women: rural poor, rural non-poor, urban poor and urban non-poor women. HPS respondents provided an estimate of the proportion of women having an abortion by method (surgical, misoprostol and other) and by provider type (doctor, nurses, pharmacists, traditional providers, woman themselves) for each of the four sub-groups of women. The likelihood of women experiencing an abortion complication was asked for each type of abortion (by provider and method) for women in each sub-group. We then multiplied the percent of women having complications for each method and provider by the distribution of women across abortion types within each sub-group.

We summed the proportion of women experiencing complications by each provider to get an estimate of the proportion that would result in a complication by method. We then adjusted this estimate of complications by the method-specific sub-group estimated proportion of women that were likely to receive treatment. Taking the sum of the method-specific numbers provides an estimate of the proportion of women who receive treatment for complications from induced abortions within each sub-group. We then weighted these estimates by the population of women in each sub-group from the ZNSA and ZDHS [2,29]. This provided us with regional estimates of the proportion of all women having abortions who receive care for complications. The inverse of this number results is the regional multiplier. A higher multiplier can result when complication rates are low or when women are less likely, or able, to seek PAC treatment. The full equation for calculating the multiplier (and the full AICM equation) is provided in S1 Appendix, and summarized in Fig 1.

The multiplier was calculated excluding respondents who rated themselves in the lowest 10% on the ten-point scale of self-reported certainty for groups of questions (e.g., urban, rural, misoprostol urban, misoprostol rural) to account for the fact that not all respondents were equally knowledgeable about all types of abortions across all areas. On the ten-point scale, the bottom 10% cutoff for self-reported certainty was four for question on rural areas, five for questions on urban areas, five for questions on misoprostol abortions in urban areas, and three for questions on misoprostol abortions in rural areas. Censoring the least certain respondents resulted in a 0.1 increase in the multiplier; this adjustment did not have a large impact on the multiplier calculation.

#### (4) Estimating the rate of induced abortion

The national total of induced abortions is the product of the multiplier and the annual PAC caseload. However, one further adjustment was made after this step to calculate the total number of abortions in 2016. Zimbabwe borders three countries with broader access to safe and legal abortions: South Africa, Mozambique and Zambia [32]. With existing high migratory rates of Zimbabwean women to neighboring countries [33], especially South Africa, it is therefore possible that women are traveling to neighboring countries to access safe abortions. To account for this, we asked HPS respondents to estimate the proportion of women in their province who travel outside of the country to have an abortion. We then took these estimates for each province, weighted by the population of women in each province, to calculate the percent of abortions occurring outside of the country by region. We added the estimated number of abortions occurring outside the country to the national total to produce the overall estimate of the number of abortions in 2016.

The regional and national abortion rates for 2016 were calculated per 1,000 women of reproductive age, and the abortion ratio per 100 live births. Upper and lower bounds were calculated for the number of abortions, abortion rate, and abortion ratio using a bootstrapping approach to estimate bounds around the multiplier. Since the HPS is a purposive rather than random sample, bootstrapping does not result in a 95% confidence interval around the mean estimate. However, given the nature of the sample and the potential of sensitivity around which respondents were selected to participate, we wanted to account for how sensitive the results are to the selected respondents. To do so, we took 10,000 draws with replacement from the HPS respondents, and calculated a multiplier after each draw. The number of draws was determined by the convergence of the estimated mean and standard errors. The upper and lower bounds presented around the multiplier contain 95% of the multiplier values estimated in the bootstrapping simulation.

#### (5) Estimating unintended pregnancy

Knowing the number of abortions, we were able to estimate the number and rate of pregnancies in Zimbabwe and the proportion of pregnancies that are unintended. Data on intention status of births comes from the 2015 ZDHS, in which women reported on whether births in the last five years were planned [2]. We assumed that all abortions were of unintended pregnancies, which is likely given that despite its legality, it is difficult to obtain an abortion for a fetal anomaly. Unintended pregnancies are comprised of all unplanned births, abortions, and miscarriages, which are estimated to be equal to 20% of the unplanned births and 10% of the abortions [34]. Intended pregnancies include planned births and miscarriages, estimated to be 20% of those births. We used these data to calculate the proportion of all pregnancies that are unintended, and the distribution of all pregnancies by intention and outcome.

## Results

### Provision of post-abortion care

The vast majority (85%) of PAC cases treated in facilities with PAC capacity in Zimbabwe were in public facilities, with half of public facility PAC cases treated at district hospitals (50%) and one-third at central hospitals (35%) (Table 2). After including the out-of-sample caseloads from the MoHCC (a total 2,239 PAC cases), we estimated a total of 25,245 PAC cases were seen in facilities in Zimbabwe in 2016 (Table 2).

**Table 2:** Annual post-abortion care caseloads by facility type and public out-of-sample facilities, by region and nationally, Zimbabwe 2016.

	National	Regions^a^		
		Matabeleland and Bulawayo	Mashonaland and Harare	South Eastern Region
**Annual PAC caseload among facilities with PAC capacity, total and by facility type** ^b^	23,006	6,607	11,976	4,423
Primary health centers	312	97	-	214
District hospitals	9,698	3,475	3,686	2,537
Provincial hospitals	2,751	787	750	1,214
Central hospitals	6,757	1,358	5,399	-
Private hospitals	2,048	406	1,304	338
NGO	1,440	484	837	120
**Annual out-of-sample public facility caseloads**				
Out-of-sample public facilities with no PAC capacity (n = 822)	14,268	3,072	7,134	4,062
Average out-of-sample public facilities referral rates	90%	81%	96%	89%
Finalized out-of-sample public facilities caseload (with adjustment)^c^	1,943	857	457	628
Missed district hospitals (n = 3)	228	24	204	-
Average district hospital referral rate	2%	0%	3%	-
Finalized MoHCC district hospital caseload (with adjustment)^c^	296	32	264	-
**Total Annual PAC Caseloads**	25,245	7,496	12,697	5,051

PAC = post-abortion care

- = Not applicable because facilities of this type with PAC capacity are not present in this region.

^a^ Regions: Matabeleland (Matabeleland North, Matabeleland South, Midlands) and Bulawayo; Mashonaland (Mashonaland East, Mashonaland West, Mashonaland Central) and Harare; South Eastern Region (Manicaland and Masvingo).

^b^ Calculated from the Health Facilities Survey and Prospective Morbidity Study.

^c^ The underreporting adjustment was derived from the data from facilities that were both in our sample and had MoHCC M&E data. The MoHCC data was consistently lower than the HFS past month, HFS typical month and PMS monthly caseload data collected. Therefore, we determined the adjustment ratio from the ratio of HFS past month divided by the MoHCC corresponding monthly caseload. The adjustments for level 1 facilities was 1.32 and 1.24 for district hospitals.

Over half (55%) of health facilities in Zimbabwe reported stock-outs of misoprostol, an essential PAC medicine, in the past three months (Table 3). Misoprostol stock-outs were reported in all central hospitals (100%), almost all primary health centers (93%), three-quarters of provincial hospitals (75%) and half of all district hospitals (50%). Over one-third of facilities also reported stock-outs for blood transfusion (35%) and IV antibiotics (34%). Half of PAC-providing facilities in Zimbabwe that should be providing MVA were not equipped with functional MVA kits (49%) (Table 3). The most commonly used PAC procedure in facilities was evacuation by curettage/D&C (61%), followed by misoprostol (18%), MVA (13%), and oxytocin (8%) (Table 3). Evacuation by curettage/D&C was the only procedure used in provincial hospitals and was the most commonly used in central (80%), district (86%) and private (90%) hospitals. One-fifth of public facilities asked patients to pay prior to being treated for PAC (Table 3).

**Table 3:** Factors that impact the capacity of facilities to provide post-abortion care, by facility type, Zimbabwe 2016.

	Total	Facility Type					
		Primary health center	District hospital	Provincial hospital	Central hospital	Private hospital	NGO
**Facility level capacity for providing post-abortion care (PAC)** ^a^							
Facilities reporting stock-outs in past three months (%)							
Misoprostol	55%	93%	50%	75%	100%	26%	18%
Blood for transfusions	35%	-	46%	38%	60%	27%	12%
IV antibiotics	34%	29%	53%	50%	80%	16%	3%
Facilities not equipped with functional MVA kits (%)	49%	-	59%	25%	0%	59%	24%
Among those providing PAC, most commonly used procedure for PAC							
Evacuation by curettage/D&C	61%	-	86%	100%	80%	90%	17%
MVA	13%	-	2%	0%	20%	3%	67%
Misoprostol	18%	55%	10%	0%	0%	7%	17%
Oxytocin	8%	45%	1%	0%	0%	0%	0%
Facilities where patients are asked to pay prior to being treated for PAC (%)	20%	5%	26%	25%	80%	-	-
Number of facilities (unweighted)	227	59	89	8	5	32	34

^a^ Health Facilities Survey

- Not applicable: According to national guidelines, primary health centers do not have the capacity to store blood, provide blood transfusions, or perform abortions or PAC using MVA, evacuation by curettage or D&C.

### Complications and treatment for induced abortion

**Fig 2:**
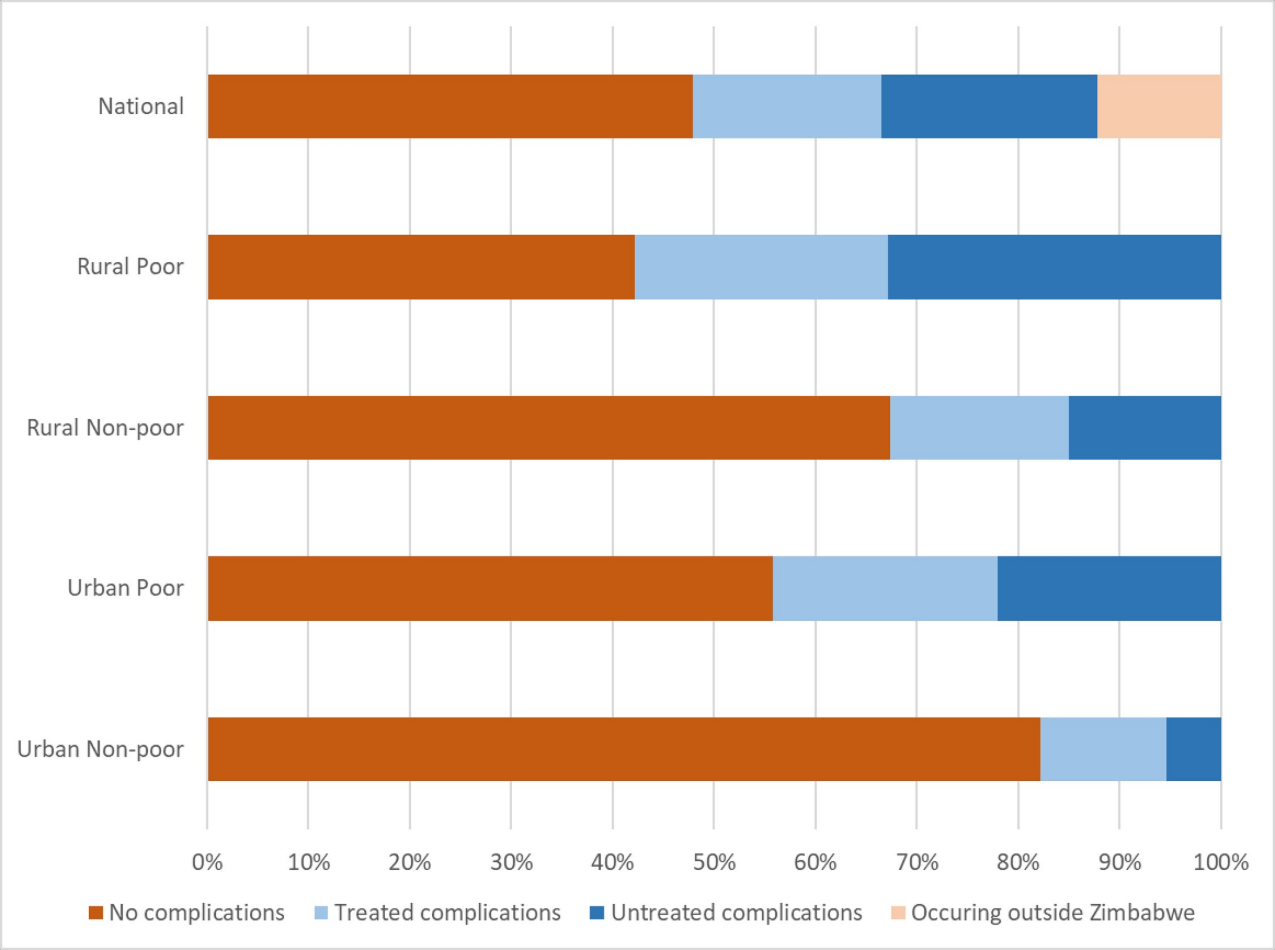
Breakdown of induced abortions by complication and treatment status, nationally and by subgroup, Zimbabwe 2016.

HPS respondents estimated that 19% of women having abortions in Zimbabwe had a complication that received treatment, while 21% had untreated complications and 48% had abortions without any complications (Fig 2). The remaining 12% of women were estimated to have had induced abortions occurring outside of Zimbabwe in neighboring countries (most likely South Africa). The probability of having untreated complications varies by residence and poverty: rural poor women were perceived to experience the highest proportion of untreated induced abortion complications (33%) compared to just 5% of urban non-poor women, 15% of rural non-poor and 22% of urban poor women (Fig 2).

The inverse of the proportion treated for complications provides the regional multipliers. For every one PAC case, there were 3.8 (uncertainty interval (UI): 3.0–4.8) additional abortions occurring in Matabeleland and Bulawayo, 4.5 (UI: 3.6–6.6) in South Eastern, and 5.4 (UI: 4.4–6.7) in Mashonaland and Harare (Table 4).

**Table 4:** Annual number of abortion complications and induced abortions, by region and nationally, Zimbabwe 2016.

	National		Regions^a^					
			Matabeleland and Bulawayo		Mashonaland and Harare		South Eastern Region	
**Total annual PAC caseload numbers**	** **	** **	** **	** **	** **	** **	** **	** **
Women receiving PAC treatment^b^ *(step 1b in Fig 1)*	25,245		7,496		12,697		5,051	
Women receiving treatment for second trimester miscarriages ^c^ (*step 1c)*	12,844		3,654		6,338		2,851	
Women treated for induced abortion complications in facilities (PAC cases—treated miscarriages) (*step 1d*)	12,401		3,842		6,359		2,200	
Treatment rate for abortion complications (per 1,000 women ages 15–49)								
All abortions	6.7		7.0		7.1		5.5	
Induced abortions	3.3		3.6		3.6		2.4	
**Total annual number of induced abortions**	** **	** **	** **	** **	** **	** **	** **	** **
Multiplier (medium estimate) (*step 2d*)	-		3.8		5.4		4.5	
			(3.0)	4.8)	(4.4)	6.7)	(3.6)	6.6)
Abortions performed outside of Zimbabwe (%)^d^ (*step 3)*	-		21%		11%		13%	
**Total number of induced abortions (*step 4*)**	**66,847**		**17,463**		**38,083**		**11,301**	
	(54000)	86171)	(14114)	22330)	(31026)	47509)	(8861)	16332)
**Abortion Rate per 1,000 women aged 15–49**	**17.8**		**16.4**		**21.3**		**12.4**	
	(14.4)	22.9)	(13.2)	20.9)	(17.4)	26.6)	(9.7)	17.9)
**Abortion Ratio per 100 live births**	**13.6**		**13.7**		**15.7**		**9.2**	

^a^ Regions: Matabeleland (Matabeleland North, Matabeleland South, Midlands) and Bulawayo; Mashonaland (Mashonaland East, Mashonaland West, Mashonaland Central) and Harare; South Eastern Region (Manicaland and Masvingo).

^b^ Includes miscarriages and induced abortions. (Source: Health Facilities Survey, Prospective Morbidity Survey and Ministry of Health and Child Care (MoHCC) Data).

^c^ Miscarriages at 13–22 weeks gestation, based on clinical data on miscarriage rates [31], calculated as 3.41 percent of all live births among women aged 15–49. There were an estimated 16,280 second trimester miscarriages in 2016. The proportion of women treated for second trimester miscarriages is from the Health Professional Survey. This estimated that 76% of women received treatment for second trimester miscarriages nationally, and these estimates ranged regionally from 68% in the South Eastern region, to 77% in Mashonaland and Harare, and 84% in Matabeleland and Bulawayo.

^d^ The percent of abortions performed outside of Zimbabwe was taken from the Health Professional Survey's estimate of proportion of abortions that occur outside of the country. The national average is 12% but we applied region specific estimates.

### Induced abortion rate and ratio

Of the 25,245 PAC cases in Zimbabwe, 12,843 were estimated to be women treated for second trimester miscarriages, resulting in an estimated 12,401 PAC cases due to induced abortions treated in facilities annually (Table 4). This translates to a treatment rate of 3.3 women treated with PAC for induced abortions per 1,000 women age 15–49 (Table 4).

After applying regional multipliers to the number of treated induced abortion complications and including the estimated number of Zimbabwean women who travel outside the country for an abortion, the total number of abortions in Zimbabwe in 2016 was 66,847 (UI: 54,000–86,171, Table 4). The abortion rate in Zimbabwe in 2016 was 17.8 induced abortions per 1,000 women of reproductive age (UI: 14.4–22.9) (Table 4). The abortion rate was the highest in Mashonaland and Harare at 21.3 per 1,000 women (UI: 17.4–26.6) and lowest in the South Eastern region at 12.4 per 1,000 women (UI: 9.7–17.9). The abortion ratio is an indicator of the likelihood of a pregnancy ending in abortion rather than a live birth. In 2016, there were 14 induced abortions per 100 live births nationally (Table 4).

### Unintended pregnancy

There were an estimated 665,447 pregnancies in Zimbabwe in 2016, resulting in a pregnancy rate for Zimbabwe of 176.8 per 1,000 women of reproductive age (Table 5). The estimated national unintended pregnancy rate is 70.4 per 1,000 women of reproductive age. The South Eastern region had the lowest unintended pregnancy rate (61.6) and Mashonaland and Harare had the highest (74.3) (Table 5).

**Table 5:** Pregnancy rate, pregnancy intentions and outcomes, by region and nationally, Zimbabwe 2016.

		Regions ^a^		
	National ^b^	Matabeleland and Bulawayo	Mashonaland and Harare	South Eastern Region
**Pregnancy rates and outcomes**	** **	** **	** **	** **
Pregnancy rate (per 1,000 women 15–49)	176.8	161.6	186.1	176.5
Unintended pregnancy rate (per 1,000 women 15–49)	70.4	71.6	74.3	61.6
Number of unintended pregnancies	264,855	76,268	132,737	56,086
**Pregnancy distribution by outcome and intention**				
Unintended pregnancies that end in abortions	10%	10%	11%	7%
Unintended pregnancies that end in births	24%	28%	23%	23%
Unintended pregnancies that end in miscarriages	6%	7%	6%	5%
Intended pregnancies that end in births	50%	46%	50%	54%
Intended pregnancies that end in miscarriages	10%	9%	10%	11%

^a^ Regions: Matabeleland (Matabeleland North, Matabeleland South, Midlands) and Bulawayo; Mashonaland (Mashonaland East, Mashonaland West, Mashonaland Central) and Harare; South Eastern Region (Manicaland and Masvingo).

^b^ National totals will not equal the exact sum of the regional numbers in order to align with published DHS measures at the national level.

Overall, 40% of pregnancies in Zimbabwe were unintended, and one-quarter of all unintended pregnancies ended in abortion (10% unintended pregnancies that end in abortion/40% unintended pregnancies) (Table 5). The percentage of unintended pregnancies ending in abortion ranged from 20% in the South Eastern Region to 29% in Mashonaland and Harare. Among all pregnancies in Zimbabwe, half (50%) ended in intended birth, 24% in unintended birth, 16% in miscarriage, and 10% ended in abortion (Table 5).

## Discussion

This study provides the first national estimate of induced abortion in Zimbabwe. Approximately 66,847 induced abortions occurred in Zimbabwe in 2016, and the national abortion rate is 17.8 abortions per 1,000 women of reproductive age. Overall, about 40% of pregnancies were unintended, of which a quarter ended in abortion. Zimbabwe has one of the lowest abortion rates in sub-Saharan Africa, likely due to high contraceptive use and a robust family planning program. It is lower than the regional estimate for Eastern Africa (34 per 1000) [35]; within sub-Saharan Africa, the only country with a comparable abortion rate is Senegal, with 17 abortions per 1,000 women of reproductive age [24]. Other countries in sub-Saharan Africa have higher abortion rates, ranging from 25 per 1,000 women in Rwanda [23], to 48 per 1,000 women in Kenya [27]. However, even with a strong family planning program and a low abortion rate, 40% of pregnancies are still unintended, suggesting that more needs to be done to help women have children if and when they want them.

Despite the low abortion rate, abortion in Zimbabwe likely contributes to the high levels of maternal morbidity and mortality as a result of both unsafe abortion and critical gaps in the provision of PAC. Providing complete PAC treatment is important for reducing maternal morbidity and mortality due to unsafe abortion. Over half of all facilities reported stock-outs of MVA equipment and misoprostol, which are safer and less expensive treatment options for managing incomplete abortions compared to D&C [36]. Central hospitals, which are located in urban areas and are intended to treat the most severe cases, all experienced critical stock-outs in the previous three months: all five central hospitals had a stock-out of misoprostol, four lacked IV antibiotics, and three lacked blood for transfusions. This is an unexpected finding; it is possible that efforts to improve rural and primary care may have come at the expense of maintaining the same standards of care in urban areas and central hospitals.

These stock-outs, along with training and staff-capacity, might also explain why evacuation by curettage/D&C remains the most common method for managing incomplete abortions (61%), despite this practice being contrary to international best practices and MoHCC guidelines [12,36], and the majority of PAC patients (65%) being in the first trimester when an MVA or misoprostol could more appropriately manage the complications [37]. The continued reliance on evacuation by curettage/D&C is likely leading to longer hospital stays, higher costs, and exposes women to greater risk [38].

There are some important limitations to this study. First, in the HFS,providers may be unable to differentiate between complications that require treatment from incomplete abortions that would resolve on their own. This has the potential to bias our abortion estimate upward, as we may be overestimating the number of PAC cases due to abortion complications. In the HPS, if respondents were able to asses correctly that these incomplete abortions do not require medical care, then multiplier would be lower. This would counteract the potential inflation of abortion complications by HFS providers, but we are unable to quantify the accuracy of both HFS and HPS respondent’s answers. While this means that there is greater uncertainty around the estimated number of women receiving PAC due to induced abortion, it is unlikely to have a large impact on the estimate of abortion incidence.

A second limitation of this study is the assumption required to remove PAC cases due to miscarriage from the estimate of induced abortion. The estimated likelihood of second trimester miscarriage (13–21 completed weeks of pregnancy) comes from US clinical data because no better source exists. This is likely a conservative estimate of the risk of miscarriage in Zimbabwe since miscarriage rates may be higher in settings with more communicable diseases, malnutrition, and the physical burdens of labor and housework. In addition, we assume that only women with second trimester miscarriages will come for treatment, but it is possible that women having miscarriages prior to 13 weeks seek facility-based care even if it is not medically indicated. However, there is no appropriate data on utilization of care for first trimester miscarriages in sub-Saharan Africa. If we underestimated the number of miscarriages because of these assumptions, the abortion rate would be an overestimate.

A third limitation of this paper is that the AICM may not fully capture safer out-of-facility abortions that occur with increasing access to misoprostol. While efforts were made to select respondents who have knowledge of out-of-facility abortions, we are not able to assess the extent to which we are accurately capturing misoprostol use. Two ways we attempted to address this were by asking HPS respondents specifically about misoprostol use, and by censoring HPS respondents’ misoprostol answers who had low self-reported certainty on these questions. While these are important advancements in the AICM, other methods for measuring abortion incidence may be necessary as misoprostol use increases.

In Zimbabwe, where abortion is restricted and highly stigmatized, there are no other data sources to estimate abortion incidence, thus the AICM is a critical advancement in contributing to what we know about induced abortion. This paper also includes some important methodological advancements to the AICM. The AICM relies on expert interviews in the HPS, but it is difficult to assess the quality of responses. Although the sample is purposive, respondents may have been included without possessing the requisite knowledge. To adjust for this possible error, this paper uses respondent’s self-reported assessments of certainty to censor respondents who were less certain about their knowledge across different groups of questions. Censoring led to a minimal change in the multiplier (0.1), but this approach should be adopted in future AICM studies as the impact of censoring may vary across HPS samples. This approach can also be used to assess and adjust for the quality of selected respondents. However, it cannot correct for respondents who have poor knowledge but high self-reported certainty. A second methodological advancement of this study is the adaptation of bootstrapping to account for potential uncertainty in the multiplier. While these are not strict confidence intervals, they are an attempt to capture some level of uncertainty in the estimated multiplier. Together, censoring and bootstrapping provide a quality check and uncertainty assessment that improve the rigor the HPS results.

### Recommendations

The gaps we find in the health care system suggest some key policy and program changes which could improve PAC services and reducing abortion-related maternal morbidity and mortality in Zimbabwe. First, misoprostol availability and training for lower level facilities will allow them to provide safer and less expensive treatment, reduce the pressures on the health system, and reduce the delays and burdens for women seeking care. Second, ensuring availability and functionality of MVA kits and training more providers in MVA will reduce the current reliance on evacuation by curettage/D&C, and, in the long term, reduce hospitalization time and costs for women and the facilities. Third, the disparity in stock-outs across facilities in urban and rural areas needs to be addressed. Improving the capacity of the central hospitals, which have the highest levels of stock-outs, is essential to improving national PAC provision. Fourth, PAC is meant to be free and must be made free. Greater steps, therefore, must be taken to fully implement the National Guidelines for Comprehensive Abortion Care in Zimbabwe [12].

There are also key health care interventions that can be taken in Zimbabwe to reduce the burden of unsafe abortion on women, their families and the health care system. Reducing unmet need for modern contraception is an essential step to reduce unintended pregnancies, which in turn reduces the risk of unsafe abortion, as well as maternal morbidity and mortality more broadly. These efforts should be comprehensive and be targeted at women with the highest levels of unmet need, including sexually active adolescents. For women who have unsafe abortions or who seek care after using misoprostol, the health care system must be able to provide essential PAC medicines and equipment at all levels and locations of facilities. To reduce unsafe abortions overall, knowledge and use of the current provisions under which abortion can be legally provided must be disseminated. Only 19% of health care providers and 42% of HPS respondents knew the four main conditions under which abortion is legal in Zimbabwe [39]. This massive gap in knowledge shows the importance of ensuring that health care providers and the general public are made aware of the provisions under which they can currently perform as well as access legal abortion. Finally, the most effective way to reduce unsafe abortion at a population level is to liberalize the abortion law. A majority of key stakeholders in the HPS (71%) suggest that liberalizing the abortion law would reduce unsafe abortion in Zimbabwe [39].

This study provides the first national estimates of abortion, unintended pregnancy, and the provision of PAC services in Zimbabwe. These findings are critical for designing evidence-based responses to prevent unintended pregnancies, improve abortion-related services, and expand access to legal and safe abortion. Such efforts are essential to reduce maternal morbidity and mortality, and to improving the lives of women and their families in Zimbabwe.

## Supporting information

S1 File Health Facilities Survey questionnaire. (PDF)

S2 File Prospective Morbidity Survey questionnaire. (PDF)

S3 File Health Professionals Survey questionnaire. (PDF)

S4 File Health Professionals Survey questionnaire visual aid. (DOCX)

S1 Appendix Appendix A: Mathematical appendix for the abortion Incidence Complications Methodology (AICM). (DOCX)

S2 Appendix Appendix B: Removing referrals from estimated caseloads by data source. (DOCX)

S3 Appendix Appendix C: Estimated post-abortion care caseloads by data source. (DOCX)
