# Correction: Abortion in Zimbabwe: A national study of the incidence of induced abortion, unintended pregnancy and post-abortion care in 2016

**DOI:** 10.1371/journal.pone.0217735

**Published:** 2019-05-30

**Authors:** Elizabeth A. Sully, Mugove Gerald Madziyire, Taylor Riley, Ann M. Moore, Marjorie Crowell, Margaret Tambudzai Nyandoro, Bernard Madzima, Tsungai Chipato

The authors discovered a data transcription error that led to an incorrect data input for the estimated number of births occurring in Zimbabwe in 2016. The authors have corrected this error and revised Tables 4 and 5. This affected the estimated total number of abortions (66,847 corrected to 65,259), the abortion rate (17.8 per 1,000 women age 15–49 corrected to 17.3 per 1,000) and the abortion ratio (14 per 100 live births corrected to 13 per 100 live births). The authors state that this does not meaningfully change the outcomes or results of the study.

In the Results subsection of the Abstract, there is an error in the second sentence. The correct sentence is: Approximately 65,259 induced abortions (uncertainty interval (UI): 52,720–84,113) occurred in Zimbabwe in 2016, which translates to a national rate of 17.3 (UI: 14.0–22.4) abortions per 1,000 women 15–49.

There are errors in the values in Table 4, “Annual number of abortion complications and induced abortions, by region and nationally, Zimbabwe 2016” and Table 5 “Pregnancy rate, pregnancy intentions and outcomes, by region and nationally, Zimbabwe 2016.” Please view the correct Table 4 and Table 5 below.

**Table 4:** Annual number of abortion complications and induced abortions, by region and nationally, Zimbabwe 2016.

	National		Regions^a^					
			Matabeleland and Bulawayo		Mashonaland and Harare		South Eastern Region	
**Total annual PAC caseload numbers**	** **	** **	** **	** **	** **	** **	** **	** **
Women receiving PAC treatment^b^ *(step 1b in Fig 1)*	25,245		7,496		12,697		5,051	
Women receiving treatment for second trimester miscarriages^c^ *(step 1c)*	13,138		3,738		6,484		2,917	
Women treated for induced abortion complications in facilities (PAC cases—treated miscarriages) *(step 1d)*	12,107		3,759		6,214		2,134	
Treatment rate for abortion complications (per 1,000 women ages 15–49)								
All abortions	6.7		7.0		7.1		5.5	
Induced abortions	3.2		3.5		3.5		2.3	
**Total annual number of induced abortions**	** **	** **	** **	** **	** **	** **	** **	** **
Multiplier (medium estimate) *(step 2d)*	-		3.8		5.4		4.5	
			(3.0)	4.8)	(4.4)	6.7)	(3.6)	6.6)
Abortions performed outside of Zimbabwe (%)^d^ *(step 3)*	-		21%		11%		13%	
**Total number of induced abortions *(step 4)***	**65,259**		**17,082**		**37,212**		**10,965**	
	(52720)	84113)	(13806)	21843)	(30317)	46423)	(8597)	15846)
**Abortion Rate per 1,000 women aged 15–49**	**17.3**		**16.0**		**20.8**		**12.0**	
	(14.0)	22.4)	(13.0)	20.5)	(17.0)	26.0)	(9.4)	17.4)
**Abortion Ratio per 100 live births**	**12.9**		**13.1**		**15.0**		**8.7**	

^a^ Regions: Matabeleland (Matabeleland North, Matabeleland South, Midlands) and Bulawayo; Mashonaland (Mashonaland East, Mashonaland West, Mashonaland Central) and Harare; South Eastern Region (Manicaland and Masvingo).

^b^ Includes miscarriages and induced abortions. (Source: Health Facilities Survey, Prospective Morbidity Survey and Ministry of Health and Child Care (MoHCC) Data).

^c^ Miscarriages at 13–22 weeks gestation, based on clinical data on miscarriage rates [31], calculated as 3.41 percent of all live births among women aged 15–49. There were an estimated 17,206 second trimester miscarriages in 2016. The proportion of women treated for second trimester miscarriages is from the Health Professional Survey. This estimated that 76% of women received treatment for second trimester miscarriages nationally, and these estimates ranged regionally from 68% in the South Eastern region, to 77% in Mashonaland and Harare, and 84% in Matabeleland and Bulawayo.

^d^The percent of abortions performed outside of Zimbabwe was taken from the Health Professional Survey's estimate of proportion of abortions that occur outside of the country. The national average is 12% but we applied region specific estimates.

**Table 5:** Pregnancy rate, intentions and outcomes, by region and nationally, Zimbabwe 2016.

		Regions ^a^		
	National ^b^	Matabeleland and Bulawayo	Mashonaland and Harare	South Eastern Region
**Pregnancy rates and outcomes**	** **	** **	** **	** **
Pregnancy rate (per 1,000 women 15–49)	180.0	164.6	189.3	179.8
Unintended pregnancy rate (per 1,000 women 15–49)	71.1	72.4	74.9	62.3
Number of unintended pregnancies	267,497	77,158	133,863	56,718
**Pregnancy distribution by outcome and intention**				
Unintended pregnancies that end in abortions	10%	10%	11%	7%
Unintended pregnancies that end in births	24%	28%	23%	23%
Unintended pregnancies that end in miscarriages	6%	7%	6%	5%
Planned pregnancies that end in births	50%	47%	50%	54%
Planned pregnancies that end in miscarriages	10%	9%	10%	11%

^a^ Regions: Matabeleland (Matabeleland North, Matabeleland South, Midlands) and Bulawayo; Mashonaland (Mashonaland East, Mashonaland West, Mashonaland Central) and Harare; South Eastern Region (Manicaland and Masvingo).

^b^ National totals will not equal the exact sum of the regional numbers in order to align with published DHS measures at the national level.

There are errors in the Induced abortion rate and ratio and the Unintended pregnancy subsections of the Results.

The correct Induced abortion rate and ratio subsection is: Of the 25,245 PAC cases in Zimbabwe, 13,138 were estimated to be women treated for second trimester miscarriages, resulting in an estimated 12,107 PAC cases due to induced abortions treated in facilities annually (Table 4). This translates to a treatment rate of 3.2 women treated with PAC for induced abortions per 1,000 women age 15–49 (Table 4). After applying regional multipliers to the number of treated induced abortion complications and including the estimated number of Zimbabwean women who travel outside the country for an abortion, the total number of abortions in Zimbabwe in 2016 was 65,259 (UI: 52,720–84,113, Table 4). The abortion rate in Zimbabwe in 2016 was 17.3 induced abortions per 1,000 women of reproductive age (UI: 14.0–22.4) (Table 4). The abortion rate was the highest in Mashonaland and Harare at 20.8 per 1,000 women (UI: 17.0–26.0) and lowest in the South Eastern region at 12.0 per 1,000 women (UI: 9.4–17.4). The abortion ratio is an indicator of the likelihood of a pregnancy ending in abortion rather than a live birth. In 2016, there were 13 induced abortions per 100 live births nationally (Table 4).

The correct Unintended pregnancy subsection is: There were an estimated 677,277 pregnancies in Zimbabwe in 2016, resulting in a pregnancy rate for Zimbabwe of 180 per 1,000 women of reproductive age (Table 5). The estimated national unintended pregnancy rate is 71.1 per 1,000 women of reproductive age. The South Eastern region had the lowest unintended pregnancy rate (62.3) and Mashonaland and Harare had the highest (74.9) (Table 5). Overall, 40% of pregnancies in Zimbabwe were unintended, and one-quarter of all unintended pregnancies ended in abortion (10% unintended pregnancies that end in abortion/40% unintended pregnancies) (Table 5). The percentage of unintended pregnancies ending in abortion ranged from 20% in the South Eastern Region to 28% in Mashonaland and Harare. Among all pregnancies in Zimbabwe, half (50%) ended in intended birth, 24% in unintended birth, 16% in miscarriage, and 10% ended in abortion (Table 5).

There is an error in the first sentence of the Discussion section. The correct sentence is: This study provides the first national estimate of induced abortion in Zimbabwe. Approximately 65,259 induced abortions occurred in Zimbabwe in 2016, and the national abortion rate is 17.3 abortions per 1,000 women of reproductive age.
