# Supplementary material for: Abortion in Zimbabwe: A national study of the incidence of induced abortion, unintended pregnancy and post-abortion care in 2016
Source: PLoS One. 2018 Oct 24;13(10):e0205239. doi: 10.1371/journal.pone.0205239 (PMC6200425; doi:10.1371/journal.pone.0205239)
Supplement: S1 File — (PDF) [file pone.0205239.s001.pdf]

STUDY OF ABORTION AND WOMEN'S HEALTH IN ZIMBABWE
HEALTH FACILITIES SURVEY

IDENTIFICATION **ID number** [ ][ ][ ][ ]

Time and Date of appt ______________________________

Contact telephone number ______________________________

Interviewer Instructions: Please fill in M1-M7 before going to the facility for the interview. If any of the information in M1-M4 has changed when you arrive, please fill in M9-12. At the end of the interview, please fill in M13. If the interview was incomplete or not done, fill in M14 and M15.

Facility Summary

To be filled out before interview		To be filled out after interview (if there are changes)	
M1	NAME OF HEALTH FACILITY: ______________________	M9	NAME OF HEALTH FACILITY: ______________________
M2	PROVINCE<br>A. Bulawayo B. Harare<br>C. Manicaland D. Mashonaland Central<br>E. Mashonaland East F. Mashonaland West<br>G. Masvingo H. Matabeleland North<br>I. Matabeleland South J. Midlands	M10	PROVINCE<br>A. Bulawayo B. Harare<br>C. Manicaland D. Mashonaland Central<br>E. Mashonaland East F. Mashonaland West<br>G. Masvingo H. Matabeleland North<br>I. Matabeleland South J. Midlands
M3	DISTRICT: ________________ [ ][ ]	M11	DISTRICT: ________________ [ ][ ]
M4	**TYPE OF HEALTH FACILITY**<br>**(Please circle the appropriate number.)**<br>1 CENTRAL HOSPITAL<br>2 PROVINCIAL HOSPITAL<br>3 DISTRICT HOSPITAL<br>4 MISSION HOSPITAL<br>5 RURAL HOSPITAL<br>6 PRIMARY HEALTH CENTER<br>7 PRIVATE HOSPTIAL<br>8 NGO - FOR PROFIT<br>9 NGO - NOT-FOR-PROFIT<br>10 Other:_________________________	M12	**TYPE OF HEALTH FACILITY**<br>**(Please circle the appropriate number.)**<br>1 CENTRAL HOSPITAL<br>2 PROVINCIAL HOSPITAL<br>3 DISTRICT HOSPITAL<br>4 MISSION HOSPITAL<br>5 RURAL HOSPITAL<br>6 PRIMARY HEALTH CENTER<br>7 PRIVATE HOSPTIAL<br>8 NGO - FOR PROFIT<br>9 NGO - NOT-FOR-PROFIT<br>10 Other:_________________________

M5	INTERVIEWER'S NAME	______________________________
M6	SUPERVISOR'S NAME	______________________________
M7	DATE OF INTERVIEW	[ ][ ] day [ ][ ] month [2][0][1][6] Year
M8	TIME STARTED	[ ][ ] h [ ][ ] M

Interview Summary

M13	**INTERVIEW OUTCOME (1ST)**<br>1 Completed<br>2 Incomplete<br>3 Refused<br>4 Not available for interview<br><br>**INTERVIEW OUTCOME (2ND)**<br>1 Completed<br>2 Incomplete<br>3 Refused<br>4 Not available for interview<br><br>**INTERVIEW OUTCOME (3RD)**<br>1 Completed<br>2 Incomplete<br>3 Refused<br>4 Not available for interview
M14	Why was the interview incomplete or not done?<br>1 Facility moved (specify place, if possible): ___________________________<br>2 Facility closed or not functioning<br>3 Facility reclassified to level not providing PAC<br>4 Facility does not offer PAC services<br>5 Not a health facility<br>6 Refused; specify reason _____________________________________<br>96 Other (specify) ____
M15	If facility is not functional, moved or relcassified, answer if possible:<br>a. When was this facility closed, moved or reclassified?<br>[ ][ ] Month [ ][ ][ ][ ] Year<br><br>b. Were PAC services provided at the facility?<br>1 Yes<br>2 No

Module 1: Basic Information

[Interviewer: Read:] Thank you for agreeing to participate. I will start by asking you a few questions about your background and this facility.

No.	Questions and Filters	Codes and Responses
101	*Gender of the respondent*<br>**[Interviewer: circle the category that applies to the respondent. Do not ask this question aloud.]**	1 Male<br>2 Female
102	What is your position at this facility?<br><br>**[Interviewer: circle the category that applies to the respondent.]**	1 OB/GYN<br>2 General practitioner<br>3 Clinical officer<br>4 Sister in charge<br>5 Midwife<br>6 Registered General Nurse (RGN)<br>7 Primary Care Nurse<br>96 Other E.g SCN (specify): ________
103	What year were you born?	☐☐☐☐
104	How many years have you worked in your current profession?<br>**[If less than 6 months, enter "00". If more or equal to 6 months but less than 1 year, enter "01".]**	☐☐ YEARS
105	How many years have you worked in this department of this facility?<br>**[If less than 6 months, conclude the interview and find someone else who is eligible.]**	☐☐ YEARS

Interviewer: Only proceed if 105 >=6 months

106 Which of the following services does this facility offer?

[Interviewer: Please read out each category, and write a response.]

Maternal and Child Health (MCH) services offered:

	Y	N	
A	1	2	Antenatal care
B	1	2	Delivery services
C	1	2	Postnatal care
D	1	2	Family planning services
E	1	2	HIV testing and counseling
F	1	2	ART (antiretroviral therapy) services
G	1	2	STI services
H	1	2	PMTCT services
I	1	2	Cervical cancer screening/VIAC
J	1	2	Immunization (ZEPI)
K	1	2	Adolescent friendly services
L	1	2	Rape and gender based violence services
M	1	2	Other gynaecological services (specify): ________
N	1	2	Other maternal health services (specify): ________

107	I am going to read out some different items that you may have in this facility. Please let me know which items are available and functional, available, or not available.<br><br>1=Available and functional<br>2=Available<br>3=Not available<br><br>**[Interviewer: Please read out each category, and write a response.]**		A&F	A	NA			
		A	1	2	3	Operating room		
		B	1	2	3	CSSD/sterilizing equipment		
		C	1	2	3	Outpatient department (OPD)		
		D	1	2	3	Recovery room		
		E	1	2	3	Pharmacy		
		F	1	2	3	Laboratory		
		G	1	2	3	Blood transfusion service		
		H	1	2	3	Intensive care unit (ICU)		
		I	1	2	3	Ultra sonogram		
108	Now I am going to read out some other items that you may have in this facility. Please let me know whether these items are commonly, sometimes, rarely, or never available in this facility.<br>1=Commonly<br>2=Sometimes<br>3=Rarely<br>4=Never<br><br>**[Interviewer: Please read out each category, and write a response.]**		C	S	R	N		
		A	1	2	3	4	Local anaesthetics	
		B	1	2	3	4	General anaesthetics	
		C	1	2	3	4	Piped Water	
		D	1	2	3	4	Electricity / solar power / generator	
		E	1	2	3	4	Blood pressure machine	
		F	1	2	3	4	Intravenous fluids	
		G	1	2	3	4	Telephone or radio communication for patient services	
		H	1	2	3	4	Ambulance to transport patient to a higher level facility within 1 hour	
109	Of the following essential medicines, do you supply it for women, do you ask them to supply it for themselves, or do you not supply it at all?<br><br>**[Interviewer: Please read out each category, and write a response.]**		**Facility supplies it**		**Women supply for themselves**	**Neither**		
			**Always**	**Sometimes**				
		A	1	2	3	4	Magnesium sulphate	
		B	1	2	3	4	Oxytocin/ergometrine	
		C	1	2	3	4	Antibiotics	
		D	1	2	3	4	Analgesics	
		E	1	2	3	4	Sedatives	
		F	1	2	3	4	Misoprostol	
110	Is misoprostol currently being used for postabortion care in this facility?			1	Yes			
				2	No			
111	Which of the following essential tests are available at this facility?<br>**[Interviewer: Please read out each category, and write a response.]**		Y	N				
		A	1	2	Hemoglobin			
		B	1	2	Syphilis test			
		C	1	2	HIV test			
		D	1	2	Pregnancy test			
		E	1	2	Malaria RDT			
		F	1	2	Blood grouping			
112	Does this facility currently possess functional manual vacuum aspiration (MVA) kits?			1	Yes			
				2	No			
				96	N/A			

Module 2: Postabortion Care

Now, I would like to ask you some questions regarding ***medical care for patients treated at this facility with abortion complications, irrespective of whether the abortion was spontaneous or induced*** .

Abortion complications, as defined here, include not only the extremely serious cases such as those with sepsis or a perforated uterus, but also those cases which are termed “incomplete abortions,” which are usually identified by heavy bleeding, and which present a somewhat less severe health risk to the woman, but which, nevertheless, require treatment in a health facility. Please do not consider threatened abortions as an abortion complication. In answering the following set of questions concerning abortion complications, please keep this definition in mind.

Abortion complications do not include the expected bleeding associated with a spontaneous or induced abortion that would resolve itself on its own, and does not require treatment.

**[Interviewer: Please note that the abortion-complication questions relate to both spontaneous and induced abortions. You should reiterate this as often as possible while completing this section.
For the questions that follow, please discourage the respondent from consulting any medical records.]**

201	Does this facility provide any treatment to women with complications from abortions? **[Interviewer: make clear this includes both spontaneous and induced abortions]**			1	Yes
				2	No → **[GO TO Q404]**
202	In which areas of this health facility are patients with abortion complications treated? **[Interviewer: Please read out the list of wards that this health facility has (circle "no" for the wards that the health facility does not have).]**		Yes	No	
		a.	1	2	General outpatient clinic
		b.	1	2	Outpatient MCH clinic
		c.	1	2	Outpatient gynaecological clinic
		d.	1	2	General surgical ward
		e.	1	2	Gynaecological/Obstetric ward
		f.	1	2	PAC or Emergency Obstetric Unit
		g.	1	2	Female ward
		h.	1	2	Intensive care unit (ICU)
		i.	1	2	Emergency ward
		j.	1	2	Other ward (specify): ______
203	In your facility, are abortion patients treated as outpatients only, inpatients only, or both? **[Interviewer: If respondent is unsure, you can remind them that outpatients are not admitted and inpatients are admitted.]**			1	Outpatient only
				2	Inpatient only → **[GO TO Q206]**
				3	Both

204	During **an average month,** about how many post-abortion care patients would you estimate are treated as **<u>outpatients</u>** in this facility as a whole? Please remember to include all patients treated for abortion complications, whether they are spontaneous or induced.<br><br>**[Interviewer : Please probe to elicit a response for an average month; if respondent is <u>not</u> able to provide you with that estimate, then probe for the number of outpatients on average per year. Specify that this is a full calendar year (i.e. from January to December). Please reiterate to the respondent that the number is for spontaneous and induced abortion patients, and should take into consideration all wards of the facility.]**	a. Number of postabortion care outpatients in an average month |__|__|__|__| <br>**OR**<br><br>b. Number of postabortion care outpatients in an average year |__|__|__|__|
205	In **the past month**, about how many post-abortion care patients were treated as **<u>outpatients</u>** in this facility as a whole? Please remember to include all patients treated for abortion complications, whether they are due to spontaneous or induced abortions.<br><br>**[Interviewer : Please probe to elicit a response for the past month; if respondent is not able to provide you with that estimate, then probe for the number of outpatients during the year 2015. Please reiterate to the respondent that the number should take into consideration all wards of the facility.]**	a. Number of postabortion care outpatients in the past month |__|__|__|__| <br>**OR**<br><br>b. Number of postabortion care outpatients last calendar year (2015) |__|__|__|__|

[Interviewer: See Q. 203. If the Health Facility provides ONLY outpatient services, go to Q. 208.]		
206	During **an average month,** about how many post-abortion care patients would you estimate were treated as **<u>inpatients</u>** at this facility as a whole? Please remember to include all patients treated for abortion complications, whether they are due to spontaneous or induced abortions.<br><br>**[Interviewer : Please probe to elicit a response for an average month; if respondent is not able to provide you with that estimate, then probe for the number of inpatients on average per year. Specify that this is a full calendar year (i.e. from January to December). Please reiterate to the respondent that the number is for spontaneous and induced abortion patients, and should take into consideration all wards of the facility.]**	a. Number of postabortion care inpatients in an average month [ ][ ][ ][ ]<br><br>**OR**<br><br>b. Number of postabortion care inpatients in an average year [ ][ ][ ][ ]
207	In t**he past month**, about how many post-abortion care patients were treated as **<u>inpatients</u>** in this facility as a whole? Please remember to include all patients treated for abortion complications, whether they are due to spontaneous or induced abortions.<br><br>**[Interviewer : Please probe to elicit a response for the past month; if respondent is not able to provide you with that estimate, then probe for the number of inpatients during the year 2015. Please reiterate to the respondent that the number should take into consideration all wards of the facility.]**	a. Number of postabortion care inpatients in the past month [ ][ ][ ][ ]<br><br>**OR**<br><br>b. Number of postabortion care inpatients last calendar year (2015) [ ][ ][ ][ ]

208	**[Interviewer : Refer to the previous figures before asking the respondent about the patient totals.]**<br><br>Just to confirm what you have told me, in an **average month** (or year), your facility treated<br><br>________ outpatients (Q204) and<br><br>________ inpatients (Q206)<br>for abortion complications.<br><br>**[Interviewer: Please read out the total number of spontaneous and induced abortion patients seen at this facility in an average month (Q 204 and Q 206).]**<br><br>Is this number correct ?<br><br>**[Interviewer: If correct, please insert again at right; if not, then correct Q 204 and Q 206 and insert at right.]**	Summary **of average** per month:<br><br>a. Outpatients (Q204) ☐☐☐☐<br><br>b. Inpatients (Q206) ☐☐☐☐<br><br>**OR**<br><br>Summary **on average** per year:<br><br>c. Outpatients (Q204) ☐☐☐☐<br><br>d. Inpatients (Q206) ☐☐☐☐
209	**[Interviewer : Refer to the previous figures before asking the respondent about the patient totals.]**<br><br>Just to confirm what you have told me, in the **past month** (or **during the year 2015),** your facility treated<br><br>________ outpatients (Q205) and<br><br>________ inpatients (Q207)<br>for a total of _____ abortion complications.<br><br>**[Interviewer: Please read out the total number of spontaneous and induced abortion patients seen at this facility last month (Q 205 and Q 207).]**<br><br>Is this number correct ?<br><br>**[Interviewer: If correct, please insert again at right; if not, then correct Q 205 and Q 207 and insert at right. Then insert this number into Q210, Q211, and Q218]**	Summary of number **in past month**:<br><br>a. Outpatients (Q205) ☐☐☐☐<br><br>b. Inpatients (Q207) ☐☐☐☐<br><br>c. Total ☐☐☐☐<br><br>**OR**<br><br>Summary of number **in year 2015**:<br><br>c. Outpatients (Q205) ☐☐☐☐<br><br>d. Inpatients (Q207) ☐☐☐☐<br><br>e. Total ☐☐☐☐

210	You told me that in the past month (or year), there were ________ PAC cases at your facility. About how many women were from this province, and how many were from another province? How many of them live outside of the country?<br><br>**[Interviewer: Make sure totals in a and b add up to total number of PAC cases in past month (or year).]**	a. Live in this province<br>b. Live in another province<br>c. Live outside of the country
211	You said that in the past month (year) there were ________ PAC patients in this facility. I want to know what types of complications these patients had.<br><br>I will read a list of complications, and after I say each complication, let me know how many of the _______ women had each symptom. Each patient may have had multiple complications. If you do not know, please give your best estimate based on your own experience working in this facility.<br><br>**[Interviewer: Read each complication aloud. The numbers must add up to at least the total number of PAC cases in the past month(year). They may add up to more than that number since a patient may suffer from more than one complication.]**	Number<br>A Incomplete abortion<br>B Incomplete evacuation<br>C Fever<br>D Sepsis<br>E Septic shock<br>F Hemorrhage<br>G Hemorrhagic shock<br>H Cervical/vaginal lacerations<br>I Uterine perforation<br>J Visceral injury (i.e. bladder, intestine)<br>X Other (specify):

The next few questions I'm going to ask you refer to the past year, 2015, rather than the past month.

212	What percentage of the post-abortion patients at this facility in the past year (2015) had complications you would classify as minor, moderate and severe? [**Interviewer: Please use the attached chart. Must total 100%]**	%<br>A Minor<br>B Moderate<br>C Severe<br>TOTAL 100%
213	What number of the post-abortion patients at this facility in the last year (2015) would you classify as near miss?<br><br>**[Interviewer: Provide this definition if respondent is unclear on the term near miss]**<br>The WHO defines "near miss" as a woman who nearly died but survived a complication that occurred during pregnancy or within 42 days of termination of pregnancy.	Number of near misses in 2015

214	a. Last year (2015), did any woman die in your facility from an abortion-related complication?<br>b. If yes, please estimate how many died.	a. 1 Yes<br>2 No<br><br>b. [ ][ ][ ] Number who died
215	What are the various treatmeants and procedures used to treat abortion-complication patients at this facility? I'm not going to read you any responses - I would like to hear what you think.<br><br>**[Interviewer: Do not prompt. Circle all that apply.]**	A Evacuation with a sharp curette (D&E, D&C)<br>B MVA (manual vacuum aspiration)<br>C Electric vacuum aspiration<br>D Surgery (e.g. laparotomy, repair injured viscera, hysterectomy)<br>E Misoprostol only (not just for cervical softening)<br>F Blood transfusion<br>G IV Antibiotics<br>H Oral antibiotics<br>I Injectable uterotonic (e.g. Oxytocin, ergometrine)<br>J IV fluids<br>X Other (specify): ______________________________
216	Of these procedures, which is the one method most commonly used for treating abortion complication patients at this facility?<br>**[Interviewer: Please mark only one response. Prompting is allowed.]**	1 Evacuation with a sharp curette (D&E, D&C)<br>2 MVA (manual vacuum aspiration)<br>3 EVA (Electric vacuum aspiration)<br>4 Misoprostol only (not just for cervical softening)<br>96 Other (specify):_____________________________
217	In the past three months, have you experienced a stock-out of any of the following drugs that impacted your ability to provide post-abortion care?<br><br>**[Interviewer: Read each item and circle yes or no for each.]**	see table below

	Y	N	
A	1	2	Misoprostol
B	1	2	Blood transfusion
C	1	2	Injectable uterotonic (e.g. Oxytocin, ergometrine)
D	1	2	IV Antibiotics
E	1	2	Oral antibiotics
X	1	2	Other PAC-related (specify): ________

[Interviewer: Go back to Q209 and add together the number of outpatients and inpatients for the past month or year. Add that number into the blank space in Q218, and then ask the question to the respondent.]

218 You had said there were _____ women treated for post abortion complications in the last month (year). I would like to ask you about the procedures used to treat these women.

In this facility, what number of women in the last month (year) were treated by the following procedures? Please only consider the primary procedure used for each woman.
[Interviewer: Read each procedure to the respondent. After you have completed the list, make sure all numbers add up to the number of women treated in the last month. There can be "0000" for certain procedures, but there must be a number in each box.]

		Number			
A	Evacuation with a sharp curette				
B	MVA (manual vacuum aspiration)				
C	EVA (Electric vacuum aspiration)				
D	Surgery only (e.g. laparatomy)				
E	Misoprostol only (not just cervical softening)				
F	Did not receive treatment				
X	Other (specify)				

NUMBER TREATED LAST MONTH (YEAR) [ ][ ][ ][ ]

219	Thinking of the women who have come into this facility in the past year with **spontaneous and induced abortion complications**: **out of every 10 women treated in this facility for abortion complications,** how many, according to your best estimate, were **19 years or younger**?<br><br>How many, according to your best estimate, were **16 years or younger**?<br><br>How many, according to your best estimate, were in the **second trimester** of pregnancy?<br><br>How many, in your opinion, had a **spontaneous abortion or miscarriage**?	a. Number **19 years of age or younger** out of every 10 PAC cases [ ][ ]<br><br>b. Number **16 years of age or younger** out of every 10 PAC cases [ ][ ]<br><br>c. Number in **second trimester of pregnancy** out of every 10 PAC cases [ ][ ]<br><br>d. Number of **spontaneous abortions** out of every 10 PAC cases [ ][ ]
220	Now I want you to think about only those women who had spontaneous abortions and came in to this facility for PAC. Out of every 10 women who have a **spontaneous abortion** treated in this facility for abortion complications**,** how many, in your opinion, were in the **second trimester**?	a. Number of spontaneous abortion cases in the **second trimester** [ ][ ]
221	How many abortion patients were referred to your facility *after having been treated* at a lower level facility in the **past month**?<br><br>Or in the year 2015?	Total number of postabortion care patients received from another health facility in the **past month** [ ][ ][ ]<br>**OR**<br>Total number of postabortion care patients received from another health facility in the **year 2015** [ ][ ][ ]
222	Why was the lower level facility not able to provide treatment?<br><br>**[Interviewer: Do not prompt. Circle all that respondent says.]**	A Lower level facility didn't have necessary equipment<br>B Lower level facility didn't have necessary personnel<br>C Lower level facility didn't have blood for transfusion<br>X Other (specify): __________<br>Y Received no referrals
223	In total, how many abortion patients have you referred to another level facility to complete treatment, **after** having treated them (either as inpatients or outpatients), in the **past month**?<br><br>Or in the year 2015?<br>[**If 00, go to Q228]**	a. Total number of patients referred for postabortion care in the past month [ ][ ][ ]<br><br>**OR**<br>b. Total number of patients referred for postabortion care in the year 2015 [ ][ ][ ]
224	Why was this facility not able to provide treatment?<br><br>**[Interviewer: Do not prompt. Circle all that apply.]**	A This facility didn't have necessary equipment<br>B This facility didn't have necssary personnel<br>C This facility didn't have blood for transfusions<br>X Other (specify):<br>__________

225	What type of facility do you refer women to?<br><br>**[Interviewer: Do not prompt. Circle all that apply.]**	A Central Hospital<br>B Provincial Hospital<br>C District Hospital<br>D Mission Hospital<br>E Rural Hospital<br>F Private Hospital<br>G NGO: For-profit<br>H NGO: Not-for-profit<br>X Other (specify) ________________________
226	How many out of every 10 women who are referred to a higher level facility do you expect will actually get to a higher level facility for treatment?	Number out of every 10 women referred for PAC services [ ][ ] **[If 10, skip to Q228]**
227	What do you think are the barriers that keep women from going to a higher level facility when they are referred to a higher level facility for treatment?<br>**[Interviewer: Do not prompt. Circle all that apply.]**	A Cannot afford travel/fees for services<br>B They do not trust the other facility<br>C They do not want their husband or family to know<br>D Lack of knowledge about severity of problem<br>E No one to accompany or support at other facility<br>X Other (specify): ______________________________
228	If a woman came to this facility for postabortion complications care, would she be asked to pay prior to being treated?	1 Yes<br>2 No
229	How much do women pay for postabortion care in this facility? It can be difficult to estimate because costs can vary widely. Can you provide what you think is the minimum and maximum paid by women in this facility?	Minimum (USD) [ ][ ][ ][ ][ ] Maximum (USD) [ ][ ][ ][ ][ ]<br><br>**[If maximum=0, skip to Q301.]**
230	Thinking about 10 women who come to this facility for treatment, how many delay receiving treatment for 48 hours or more after arriving at this facility because of the cost? Please give me your best estimate.	[ ][ ] out of 10
231	Out of 10 women who come to this facility for treatment, how many leave without receiving any treatment because of the cost?	[ ][ ] out of 10

Module 3: Postabortion Contraceptive Counseling and Services

301	Do you offer contraceptive **counseling** services to patients seen for postabortion care at this facility in the same ward in which PAC is provided?	1	Yes, always
		2	Yes, sometimes
		3	No **[Go to 304]**
302	What topics are generally covered in contraceptive counselling for women who received PAC services at this facility? **[Interviewer: Do not prompt. Multiple responses are allowed.]**	A	Instructions on correct use of methods
		B	Informed about a range of contraceptive methods
		C	Informed on effectiveness of methods
		D	Informed on side effects of methods
		E	What to do in case of incorrect use of methods (e.g. skipped pills)
		F	Emergency contraception in case of failure to use a method before or during intercourse, or because of method failure (e.g. broken condom)
		G	Benefits of contraception
		H	Abstinence
		X	Other (specify): ______________________
303	Does your facility have youth peer educators who do contraceptive counseling?	1	Yes
		2	No
		8	Don't Know
304	Do you offer contraceptive **methods** to postabortion care patients on the premises of this facility?	1	Yes
		2	No **[Go to 317]**
		8	Don't Know **[Go to 317]**

				Always	Sometimes	Rarely	Never
305	How often do you offer contraceptive services to the following groups of PAC patients --always, sometimes, rarely or never? **[Interviewer: Please read all responses and circle whether the respondent says they offer contraceptives Always, Sometimes, Rarely, or Never.]**	A	Women who have many children	1	2	3	4
		B	Women who have no children	1	2	3	4
		C	Women who are married	1	2	3	4
		D	Women who are not married	1	2	3	4
		E	Women who are HIV positive	1	2	3	4
		F	Adolescents (19 years of age or younger)	1	2	3	4
		G	Women 45 years of age or older	1	2	3	4
		H	Partners accompanying women receiving post-abortion care	1	2	3	4

306	Out of 10 women treated for abortion complications, on average how many are actually given a **method of family planning** on discharge from this facility?	☐☐ Women

307	What are all of the methods that you provide to post-abortion patients or their partners at this facility?<br><br>**[Interviewer: Do not prompt. Multiple responses are allowed]**	A	Rhythm (periodic abstinence) with calendar
		B	Male condom
		C	Female condom
		D	Pills
		E	Injectables
		F	Implants
		G	Intrauterine devices (IUD/IUCD)
		H	Female sterilization
		I	Vasectomy
		X	Other (specify): ____________
308	Are there any other methods you would like to offer that you currently do not? If so, which ones:<br><br>**[Interviewer: Do not prompt. Multiple responses are allowed.]**	A	Rhythm (periodic abstinence) with calendar
		B	Male condom
		C	Female condom
		D	Pills
		E	Injectables
		F	Implants
		G	Intrauterine devices (IUD/IUCD)
		H	Female sterilization
		I	Vasectomy
		X	Other (specify): ____________
		Y	No other methods **SKIP TO 310**
309	What are the reasons you are not offering those methods?<br><br>**[Interviewer: Do not prompt. Multiple responses are allowed.]**	A	Stock-out
		B	Lack of staff trained to provide other methods
		C	Provider preferences
		D	Client preferences
		E	Facility does not allow (e.g. faith based org)
		F	Not familiar enough with other methods
		G	Method has never been available
		X	Other (specify): ____________
**[Interviewer: Review respondent's answer to Q307.<br>If did not select any long-acting reversible methods (any of E, F, or G), GO TO 310<br><br>If answered E, F, or G: GO TO 311]**			
310	Why do you not offer some long-acting reversible methods such as injectables, implants, intrauterine devices (IUD) at this site?<br>**[Interviewer: Do not prompt. Multiple responses are allowed]**	A	Stock-out
		B	Lack of staff trained to provide certain methods
		C	Provider preferences
		D	Client preferences
		E	Facility does not allow (e.g. faith based org)
		F	Not familiar enough with methods
		G	Method has never been available
		X	Other (specify): ____________

311	Why do you think women may refuse a modern contraceptive method after having had an abortion?<br>**[Interviewer: Do not prompt. Multiple responses are allowed]**	A Woman opposed to use<br>B Partner opposed to use<br>C Family opposed to use<br>D Religious opposition<br>E Health concerns<br>F Fear of side effects<br>G Wanted more effective method<br>H Costs too much<br>I Inconvenient to use<br>J Interference with body processes<br>K Not having sex<br>L Infrequent sex<br>M Preference for traditional methods<br>N Impact of contraception on their fertility<br>O Wants to conceive<br>X Other (specify): ____________
312	Do you think that women prefer a contraceptive method they can keep private and unknown to their partner/spouse?	1 Yes<br>2 No **[SKIP TO 314]**<br>8 DK **[SKIP TO 314]**
313	Which method(s) do women prefer to use to keep their contraceptive use unknown to their partner/spouse?<br>**[Interviewer: Do not prompt. Multiple responses are allowed]**	A Rhythm (periodic abstinence) with calendar<br>B Pills<br>C Injectables<br>D Implants<br>E Intrauterine devices (IUCD/IUD)<br>F Female sterilization<br>X Other (specify): ____________
314	Of the contraceptive methods you do offer here, which methods do you recommend to adolescent girls 19 years of age or younger and their partners?<br>**[Interviewer: Do not prompt. Multiple responses are allowed]**	A Rhythm (periodic abstinence) with calendar<br>B Male condom<br>C Female condom<br>D Pills<br>E Injectables<br>F Implants<br>G Intrauterine devices (IUCD/IUD)<br>H Abstinence<br>X Other (specify): ____________<br>Z None **[Go to 401]**
315	What methods, if any, do you offer to girls 16 years of age or younger?<br>**[Interviewer: Do not prompt. Multiple responses are allowed]**	A Rhythm (periodic abstinence) with calendar<br>B Male condom<br>C Female condom<br>D Pills<br>E Injectables<br>F Implants<br>G Intrauterine devices (IUCD/IUD)<br>H Abstinence<br>X Other (specify): ____________<br>Z None **[Go to 401]**
316	a. When offering contraceptive services to adolescent girls, do you require their parents' consent?<br>b. If so, below what age?	a. 1 Yes **Fill in age below**<br>2 No<br>8 DK<br>b. **Below** [ ][ ] **years of age**

[Interviewer: STOP. If you asked Q315, continue to Section 4. You should only ask the following questions if respondent aswered question above that skipped to Q317]

317	Why do you not offer contraceptive methods on-site?<br><br>**[Interviewer: Do not prompt. Multiple responses are allowed.]**	A Providers aren't adequately trained<br>B The services are not functional<br>C The services are not integrated<br>D The methods are not available<br>E Facility does not allow (e.g. faith based org)<br>F Services are offered in another facility close by<br>G Services are offered elsewhere in the facility<br>X Other (specify) ____________
318	Do you refer postabortion care patients to other facilities that provide contraceptive services?	1 Yes, to another facility<br>2 Yes, to another section in my facility<br>3 No **[Go to 401]**
319	What type of facility do you refer women to?<br><br>**[Interviewer: Do not prompt. Multiple responses are allowed.]**	A Central Hospital<br>B Provincial Hospital<br>C District Hospital<br>D Mission Hospital<br>E Rural Hospital<br>F Private Hospital<br>G NGO: For-profit<br>H NGO: Not-for-profit<br>I ZNFPC Clinic<br>X Other (specify) ____________

Module 4: General

401	In your opinion, how could treatment for abortion complications be improved at this facility?<br><br>**[Interviwer: Do not read. Circle all that apply.]**	A	Have a private room for postabortion care patients
		B	Improve/expand infrastructure
		C	Have more people trained in misoprostol use
		D	Have more people trained in D&C
		E	Have more people trained in MVA
		F	Have more lower level facilities allowed and equipped to provide PAC
		G	Have more MVA equipment available
		H	Have more misoprostol available
		I	Have more antibiotics available
		J	Have more blood available for transfusions
		K	Have more pain medication available
		L	Provide information on contraception and give methods
		M	Copy of PAC guidelines available at this facility
		N	Provide more support to staff (e.g. refresher courses)
		O	Nothing is needed, current treatment is adequate
		P	Provide free postabortion care
		Q	Vehicle to transport patients who have been referred
		X	Other (specify):
402	Under what conditions in Zimbabwe is abortion legal?<br><br>**[Interviewer: Do not read. Multiple responses are allowed.]**	A	Don't know
		B	If the woman's physical health is at risk
		C	If pregnancy is from rape
		D	If pregnancy is from incest
		E	If the foetus is handicapped/foetal anomaly
		F	If the woman is HIV positive
		G	If the woman is mentally incapacitated
		H	If the woman's mental health is at risk
		I	If the girl or woman is still in primary or secondary school
		J	If a girl is under age 16
		K	Economic reasons (e.g. cannot care for the child)
		L	If the girl or woman is unmarried
		M	If pregnancy is from contraceptive failure
		N	If the woman doesn't want the pregnancy
		O	Under no circumstances
		X	Other (specify):

		Situations	Y	N	D/K
403	Do you think abortion should be allowed in the following situations?<br><br>**[Interviewer: Please read out each situation and circle respondent's answer: Yes, no, or don't know/DK.]**	a. if the woman's physical health is at risk	1	2	8
		b. if the woman is mentally incapacitated	1	2	8
		c. if the woman's mental health is at risk	1	2	8
		d. if the girl or woman is still in primary or secondary school	1	2	8
		e. if a girl is under age 16	1	2	8
		f. economic reasons (e.g. cannot care for the child)	1	2	8
		g. If the girl or woman is unmarried	1	2	8
		h. if pregnancy is from rape	1	2	8
		i. if pregnancy is from incest	1	2	8
		j. If pregnancy is from contraceptive failure	1	2	8
		k. if foetus is handicapped or has anomalies	1	2	8
		l. if the woman doesn't want the pregnancy	1	2	8
		m. if the woman is HIV positive	1	2	8
		x. other (specify):	1	2	8

404	Has this facility ever provided a legal abortion based on the criteria in Zimbabwe?	1 Yes<br>2 No **[Go to Q406]**<br>8 Don't know **[Go to Q406]**
405	How many times has this happened during the last 12 months or during the last 24 months?	a. During the last 12 months ☐☐☐☐<br>**OR**<br>b. During the last 24 months ☐☐☐☐
406	Are you aware of any woman coming to this facility to have a legal abortion who was denied that service by a provider in this facility?	1 Yes<br>2 No<br>8 Don't know
407	How many women has your facility referred to neighbouring countries for an induced abortion in the last year (2015)? **[Interviewer: If respondent doesn't know, then enter '999']**	☐☐☐ women in 2015

Module 5: Medical Records

[Interviewer please read:] I have finished my questions directly for you. For the following questions, it may be helpful to consult records. I want to ask you about beds in the facility, training, and recorded PAC cases and legal abortions.

[Interviewer: For the following questions, please allow the respondent to check the medical records in providing answers and go with them to check the records. This is the only section of the questionniare in which medical records should be checked, and it should only be completed once all other questionnaires sections are finished.]

501	How many beds does this facility have in total?	☐☐☐☐ BEDS
502	How many female or gynecological beds does this facility have?	☐☐☐☐ FEMALE OR GYNECOLOGICAL BEDS
503	On average about how many deliveries take place at your facility each month?<br><br>**[Interviewer: if respondent can't answer for average month, probe for the total number in 2015.]**	a. Deliveries per month ☐☐☐☐<br>**OR**<br>b. Total number of deliveries during the 2015 calendar year ☐☐☐☐
	**Questions about formal training (both pre-service and in-service)**	
504	How many people in this facility were formally trained to provide postabortion care (PAC) services?<br><br>**[Interviewer: Formal training include both pre-service and in-service]**	☐☐☐☐ STAFF
505	How many people in this facility have received formal training to use sharp curetteage?	☐☐ PEOPLE
506	How many people in this facility have received formal training to use MVA?	☐☐ PEOPLE
507	How many people in this facility have received formal training to use electric vacuum aspiration (EVA)?	☐☐ PEOPLE
508	How many people in this facility have received formal training to use misoprostol for postabortion care?	☐☐ PEOPLE

509	Do you keep records on the number of women treated for abortion complications at this facility?	1 Yes<br>2 No **[Skip to END.]**
510	How are these records kept?<br>**[If the facility keeps more than one type of record, circle the one that is the most complete and up-to-date.]**	1 Paper-based log book<br>2 Electronic, just in facility<br>3 Electronic, centralised in database outside<br>96 Other (specify): ________
511	Is there any recorded information about the number of women treated in this health facility for abortion complications that I could look at?	1 Yes<br>2 No<br><br>**IF YES:**<br>Details:<br>**Number of women treated for complications of abortion:**<br>a. In the last month:<br>[ ][ ][ ][ ] Outpatients<br>[ ][ ][ ][ ] Inpatients<br>[ ][ ][ ][ ] Total<br>OR<br>b. In the first quarter<br>[ ][ ][ ][ ] Outpatients<br>[ ][ ][ ][ ] Inpatients<br>[ ][ ][ ][ ] Total<br>OR<br>c. During the year 2015:<br>[ ][ ][ ][ ] Outpatients<br>[ ][ ][ ][ ] Inpatients<br>[ ][ ][ ][ ] Total
512	Is there any recorded information about the number of legal abortions provided in this health facility that I could look at?	1 Yes<br>2 No<br><br>**IF YES:**<br>Details:<br>**Number of legal abortions provided in this health facility**<br>a. In the last month:<br>[ ][ ][ ][ ] Outpatients<br>[ ][ ][ ][ ] Inpatients<br>[ ][ ][ ][ ] Total<br>OR<br>b. In the first quarter<br>[ ][ ][ ][ ] Outpatients<br>[ ][ ][ ][ ] Inpatients<br>[ ][ ][ ][ ] Total<br>OR<br>c. During the year 2015:<br>[ ][ ][ ][ ] Outpatients<br>[ ][ ][ ][ ] Inpatients<br>[ ][ ][ ][ ] Total

END: Thank the respondent for their time.

M16	TIME ENDED	__ __ h __ __ M

[INTERVIEWER INSTRUCTIONS: Please fill the following table ONLY if the facility has many PAC providers and no one can be found who is able to provide a combined count for all providers. In such situations, collect data on personal caseload directly from as many providers as possible and fill in the table below. Please add lines to this table as needed.]

**Provider type**	**Ward**	**PAC cases treated in the past month**		
		Outpatient	Inpatient	**Total**

Commentary: ____

Health Facilities Survey Process Completion Checklist

Please verify that each task below has been completed. If you are responsible for the task, add your signature to the corresponding task and date when the activity was completed.

	Field level				Office level	
**Questionnaire checked for:**	Interviewer	Date	Supervisor	Date	Data entry clerk signature	Date
**a. Questionnaire completeness checked**						
**b. Clear handwriting checked**						
**c. Appropriate response type checked**						
**d. Skip patterns properly followed**						

I verify that all of this information is completed.
Supervisor's signature: ____________________________

FIRST DATA ENTRY COMPLETE: ____________________________________

SECOND DATA ENTRY COMPLETE: ____________________________________
