# Supplementary material for: Abortion in Zimbabwe: A national study of the incidence of induced abortion, unintended pregnancy and post-abortion care in 2016
Source: PLoS One. 2018 Oct 24;13(10):e0205239. doi: 10.1371/journal.pone.0205239 (PMC6200425; doi:10.1371/journal.pone.0205239)
Supplement: S2 File — (PDF) [file pone.0205239.s002.pdf]

PROSPECTIVE MORBIDITY SURVEY IN ZIMBABWE, 2016

Questionnaire for Patient

University of Zimbabwe-University of California San Francisco, Zimbabwe Ministry of Health and Child Care, and Guttmacher Institute

Questionnaire ID: ☐☐☐☐☐☐☐☐

(number should be same as provider questionnaire)

	BACKGROUND INFORMATION		CODES
001	HEALTH FACILITY NAME ______________________		
002	HEALTH FACILITY CODE		☐☐☐☐
003	DATE OF INTERVIEW		☐☐ ☐☐ ☐☐ (DD MM YY)
004	DISTRICT ______________________		
005	TYPE OF HEALTH FACILITY	Central hospital	1
		Provincial hospital	2
		District hospital	3
		Mission hospital	4
		Rural hospital	5
		Primary health center	6
		Private hospital	7
		NGO - For profit	8
		NGO - Not-for-profit	9
		Other: ______________	10
006	START TIME	☐☐ hr ☐☐ min	
007	INTERVIEWER NAME ______________________		☐ Code
008	LANGUAGE OF INTERVIEW	English	1
		Shona	2
		Ndebele	3
009	RESULT OF INTERVIEW	COMPLETE	1
		REFUSED	2
		INCOMPLETE	3
		PATIENT UNABLE TO ANSWER	4
		PATIENT DIED PRIOR TO INTERVIEW	5
		OTHER (SPECIFY)______________	96
010	IF THE PATIENT WAS UNABLE TO ANSWER OR REFUSED, DO YOU THINK THEY ARE 19 YEARS OF AGE OR YOUNGER?	YES	1
		NO	2

Section I: SOCIO-DEMOGRAPHICS OF THE PATIENT

Thank you for agreeing to participate in this survey. I want to again assure you that your answers will be kept confidential. I would like to begin by asking you a few questions about yourself, your job, and your home.

	QUESTIONS	RESPONSES		
101	In what year were you born?	[ ][ ][ ][ ] Year		
102	Do you live in an urban or rural area of Zimbabwe?	Urban	1	
		Rural	2	
		From outside Zimbabwe	3	
103	What is your current marital status?	Never married/ Never lived together with a partner	1	
		With partner but not living together	2	
		Living together with partner	3	
		Married	4	
		Separated/Divorced/Widowed	5	
104	What is your religion?	Apostolic	1	
		Pentecostal	2	
		Protestant	3	
		Roman Catholic	4	
		Other Christian	5	
		Traditional religions	6	
		Muslim	7	
		None	8	
		Other	96	
		(specify) ____________		
105	Are you currently enrolled in school?	No	0	
		Yes, part-time	1	
		Yes, full-time	2	
106	What is your highest level of formal education?	No education	1	
		Did not complete primary	2	
		Completed primary	3	
		Some secondary	4	
		O-levels	5	
		A-levels	6	
		University or college	7	
		Post-graduate education	8	
107	What kind of work do you mainly do?	Work, full time	1	
		Work, part-time	2	
		Self-employed	3	
	[Read aloud and circle only one response]	Full time housewife	4	**→Skip to 109**
		Student	5	**→Skip to 109**
		Unemployed	6	**→Skip to 109**
		Unpaid family worker	7	**→Skip to 109**
		Other	96	
		(specify) ____________		

108	Are you working for pay or without pay?	Working for pay — 1<br>Working without pay — 2
109	How many people slept in your household last night, including you?	[ ][ ] members
110	In your household, do you have:<br><br>**[Interviewer: Read out each item and circle respondent's answer. Fill in the "Other" option only if the respondent gives a different response unprompted]**	(YES / NO)<br>a. Electricity? — 1 / 2<br>b. A battery/generator? — 1 / 2<br>c. A solar panel? — 1 / 2<br>d. A radio? — 1 / 2<br>e. A television? — 1 / 2<br>f. A mobile telephone? — 1 / 2<br>g. A landline? — 1 / 2<br>h. A refrigerator? — 1 / 2<br>i. A computer? — 1 / 2<br>x. Other (specify): _________________ — 96
111	Does a member of your household own:<br><br>**[Interviewer: Read out each item and circle respondent's answer.]**	(YES / NO)<br>a. A wristwatch? — 1 / 2<br>b. A bicycle? — 1 / 2<br>c. A motorcycle or scooter? — 1 / 2<br>d. An animal-drawn cart? — 1 / 2<br>e. A car or vehicle? — 1 / 2<br>f. A tractor? — 1 / 2<br>g. A boat with a motor? — 1 / 2<br>h. A wheelbarrow? — 1 / 2
112	What is the main material that your roof is made of?<br><br>**[Interviewer: Circle only the one main material.]**	Natural roofing (thatch) or no roof — 1<br>Rudimentary roofing (rustic mat/wood planks/pastic) — 2<br>Finished roofing (metal/wood/tiles/cement/asbestos) — 3<br>Other: _________________ — 96
113	What is the main source of drinking water for members of your household?<br><br>**[Interviewer: Circle only the one main source of drinking water.]**	Piped water — 1<br>Tube well or borehole — 2<br>Dug well — 3<br>Water from spring — 4<br>Rainwater — 5<br>Tanker truck — 6<br>Cart with small tank — 7<br>Surface water (river/dam/lake/pond/steam/canal/irrigation channel) — 8<br>Bottled water — 9<br>Other: _________________ — 96
114	What kind of toilet facility do members of your household usually use?<br><br>**[Interviewer: Circle only the one most common.]**	Flush or pour flush toilet — 1<br>Blair toilet — 2<br>Latrine/pit toilet — 3<br>Bucket toilet — 4<br>No faciity/bush/field — 5<br>Other: _________________ — 96

Section II: REPRODUCTIVE HISTORY OF THE PATIENT		
**Now I would like to ask you some questions about your personal life. I would like to know more about all the pregnancies that you have had during your life. I would like to remind you that your answers will be kept confidential.**		
	**QUESTIONS**	**RESPONSES**
201	How many pregnancies have you had in total, including the one you are seeking care for?	*(number)* ☐☐
202	Have you ever given birth?	Yes 1<br>No 2 →**Skip to 206**
203	In total, how many children have you given birth to (including any that may have been born alive but later died)?	*(number)* ☐☐
204	How many biological living children do you have, including those who are not currently living with you?<br>**[Interviewer: Check to make sure that this number is not higher than Q203]**	*(number)* ☐☐
205	When was your last childbirth?	☐☐ Month<br>☐☐☐☐ Year
206	Before the pregnancy that led you to seek care in this facility:<br>a. How many spontaneous abortions have you had?<br>b. How many induced abortions had you had?	a. *(number)* ☐☐<br>b. *(number)* ☐☐
207	Excluding the pregnancy for which you are seeking care in this facility, how many times have you been pregnant when you did not want to be?	*(number)* ☐☐

Section III: RECENT PREGNANCY AND DELAYS TO SEEKING CARE

Now I would like to talk about your recent pregnancy, that is, the one that resulted in the complications you are being treated for now.

	QUESTION	RESPONSES		
301	At the time you became pregnant with the last pregnancy that resulted in your seeking care at this facility, did you want to become pregnant then, did you want to wait until later, or did you not want to have any more children at all?	Wanted **then**	1	→ **skip to 303**
		Wanted **later**	2	
		Did **not want at all**	3	→ **skip to 304**
		Don't know	98	→ **skip to 304**
302	How much longer would you like to have waited before getting pregnant?	Less than 2 years	1	→ **skip to 304**
		Two or more years	2	→ **skip to 304**
		Don't know	98	→ **skip to 304**
303	Even though you wanted the pregnancy at the time you became pregnant, did you later change your mind in the weeks or months after learning that you were pregnant?	Yes	1	
		No	2	→ **skip to 305**
304	Why did you not want the pregnancy at that time?<br><br>**[Interviewer: Do not read aloud. Circle all that apply. Probe after woman is done listing responses, "Any other reasons?"]**	Have enough children	A	
		High cost of raising children	B	
		It is too soon after last birth	C	
		Would have to leave school	D	
		Not married	E	
		Would have to stop working	F	
		Got pregnant by someone other than husband/regular partner	G	
		Abandoned by partner	H	
		Forced to have sex	I	
		Health reasons	J	
		Feel too old to have children	K	
		Forced by partner to end pregnancy	L	
		Partner denied being the father	M	
		Parents/other relatives were opposed	N	
		Other<br>(specify)	X	
305	When you became pregnant this time **(the last pregnancy** that resulted in your **seeking treatment in this facility**), were you doing something or using any method to delay or avoid getting pregnant?	Yes	1	
		No	2	→ **skip to 307**
306	Which methods were you using (or what were you doing) at the time you became pregnant?<br><br>**[Interviewer: Remind the patient that we are referring to the last pregnancy that resulted in her seeking treatment in this health facility]**<br><br>**[Interviewer: Do not read out, circle as many as applicable.]**	Rhythm (periodic abstinence) with calendar	A	**SKIP TO 308**
		Male condom	B	
		Female condom	C	
		Pills	D	
		Injectables	E	
		Implants	F	
		Intrauterine devices (IUD/IUCD)	G	
		Female sterilization	H	
		Vasectomy	I	
		Withdrawal	J	
		Other Traditional method<br>(specify)	X	

No.	Question	Response	Code	Skip
307	Please tell me the reasons why you were not using a method of family planning when you became pregnant this time?<br><br>**[Interviewer: Remind the patient that we are referring to the last pregnancy that resulted in her seeking treatment in this health facility]**<br><br>**[Interviewer: Do not read aloud. Circle all that apply. When respondent stops listing, then ask:]**<br><br>Are there any other reasons why you were not using a method of family planning when you became pregnant this time?	Fear of side effects	A	**ask 307a**
		Health reasons	B	
		Husband or partner opposed	C	**ask 307b**
		Other family members opposed	D	**ask 307c**
		Don't want to use it/don't like it	E	**ask 307d**
		Wanted more children	F	**skip to 308**
		Didn't know where to get FP	G	
		FP services too far away	H	
		Against religious beliefs	I	
		Couldn't afford family planning	J	
		Didn't think I could get pregnant	K	
		Didn't expect to have sex	L	
		Didn't think about it	M	
		Coerced into having sex	N	
		I have never used family planning	O	
		Other	X	
		(specify)		
307a	Which side effects or health impacts were you concerned about?<br><br>**[Interviewer: Do not read aloud. Circle all that apply. When respondent stops listing, then ask:]**<br><br>Are there any other side effects or health impacts that concern you?<br><br>**[Interviewer: Go to 308 if any option besides C, D, or E circled in 307; if C circled, go to 307b; if D circled, go to 307c; if E go to 307d.]**	Loss of menstrual cycle	A	
		Heavy periods	B	
		Spotting (irregular bleeding)	C	
		Infertility	D	
		Weight gain	E	
		Weight loss	F	
		Mood swings	G	
		Headaches	H	
		Nausea	I	
		Fatigue	J	
		Contraindications (e.g. high blood pressure)	K	
		Other (specify): ________________ ________________	X	
307b	For what reasons is your [husband/partner] opposed to your using family planning?<br><br>**[Interviewer: Do not read aloud. Circle all that apply. When respondent stops listing, then ask:]**<br><br>Are there any other reasons your [husband/partner] is opposed?<br><br>**[Interviewer: Go to 308 if any option besides D or E circled in 307; if D circled, go to 307c; if E circled, go to 307d.]**	Loss of menstrual cycle	A	
		Heavy periods	B	
		Spotting (irregular bleeding)	C	
		Fear of infertility	D	
		Fear of weight gain	E	
		Fear of weight loss	F	
		Mood swings	G	
		He wants more children	H	
		Against religious beliefs	J	
		He thinks I am or would be unfaithful	K	
		Other (specify): ________________ ________________	X	
307c	For what reasons [is this person/are these people] opposed to your using a method?<br><br>**[Interviewer: Do not read aloud. Circle all that apply. When respondent stops listing, then ask:]**<br><br>Are there any other reasons [this person/these people] are opposed?<br><br>**[Interviewer: Go to 308 if any option besides E circled in 307; if E circled, go to 307d.]**	They want me to abstain from sex	A	
		They want me to have more children	B	
		Fear that contraception will cause infertility	C	
		Fear of contraception's side effects	D	
		Fear that contraception may interfere with bodily processes	E	
		Against their religious beliefs	F	
		Other (specify): ________________ ________________	X	

307d	For what reasons do you not want to use family planning?<br><br>**[Interviewer: Do not read aloud. Circle all that apply. When respondent stops listing, then ask:]**<br><br>Are there other reasons you do not want to use family planning?	Loss of menstrual cycle	A
		Heavy periods	B
		Spotting (irregular bleeding)	C
		Fear of infertility	D
		Fear of weight gain	E
		Fear of weight loss	F
		Fear of mood swings	G
		Fear of headaches	H
		Fear of nausea	I
		Fear of fatigue	J
		Worried it won't work	K
		Inconvenient to use	L
		Inconvenient to obtain	M
		Worried family will find out	N
		My religion does not allow it	O
		Family planning is immoral	P
		Other (specify): ____________	X
308	Do you intend to use a method in the next 12 months?	Yes	1
		No	2
		Don't know	98
309	Which methods of family planning are available to you, if you wanted to start using?<br><br>**[Interviewer: Do not read aloud. Circle ALL that apply]**	Rhythm (periodic abstinence) with calendar	A
		Male condom	B
		Female condom	C
		Pills	D
		Injectables	E
		Implants	F
		Intrauterine devices (IUD/IUCD)	G
		Female sterilization	H
		Vasectomy	I
		Withdrawal	J
		Other Traditional method	X
		(specify) ____________	
310	How many weeks (or months) pregnant were you before you experienced the loss of this pregnancy?	a. Weeks	☐☐
		OR	
		b. Months	☐☐
		Unknown gestation	98

I would now like to ask you a few questions about when you first started having medical problems associated with your most recent pregnancy. I am going to ask about the complications you experienced, and how they felt to you. It does not matter whether you are seeking treatment for a spontaneous or an induced abortion. Your honest answers to these questions will help us understand problems women face receving medical care, and how the country can better respond to them. The answers you provide will not in any way affect the treatment you receive.

			Yes			
			Mild	Moderate	Severe	No
311	Please describe any of the following health problems you had when you <u>first started having health problems</u> as a result of this pregnancy complication as a mild, moderate or severe problem.<br><br>**[Interviewer: Read out each cateory, ie "Did you experience bleeding?" If YES, ask if problem was mild, moderate, severe. Probe for any other problems]**	a. Bleeding	1	2	3	4
		b. Pain	1	2	3	4
		c. Injuries	1	2	3	4
		d. Fever	1	2	3	4
		e.Vaginal discharge (other than blood)	1	2	3	4
		f. Other (specify) ____________	1	2	3	4

312	After realizing you were having health problems, did you tell someone?	Yes	1	
		No	2	→ **skip to 314**

313	Whom did you tell?<br><br>**[Interviewer: Do not read aloud. Circle ALL that apply]**	Husband/partner/boyfriend	A
		Mother/mother-in-law	B
		Sister	C
		Some other relative	D
		Friend	E
		Health care worker	F
		Traditional health provider	G
		Other person (specify) ________	X
		**SKIP TO 315**	
314	What was (were) the reason(s) why you did not tell anybody you were experiencing health problems?<br><br>**[Interviewer: Do not read aloud. Circle ALL that apply]**	Did not consider it serious enough	A
		Afraid of husband/partner's reaction	B
		Afraid of family's reaction	C
		Afraid of being reported to police	D
		Did not want others to know	E
		Do not have a close friend/relative to tell	F
		Felt embarassed	G
		Other (specficy)	X

315 After experiencing health problems, how long did it take for you to realize you needed medical care?

[Interviewer: Please ask and indicate number of either minutes, hours, days or weeks. If they cannot give a numerical answer, circle the column for unit of time it was within]

Time from health problem to realizing needed care	Time		This unit
a. Minutes			96
b. Hours			96
c. Days			96
d. Weeks			96

316	What was it about your health problems that made you realize you needed medical care?<br><br>**[Interviewer: Do not read aloud. Circle ALL that apply]**	Symptoms would not stop	A
		Symptoms felt severe	B
		Worried about health consequences of waiting	C
		Experienced this problem before	D
		Partner/relative/friend told me to seek care	E
		Symptoms interfered with my work/school/housework	F
		Other (specify) ________	X
317	Did you seek help when you realized you needed medical care?	Yes	1
		No	2 → **skip to 319**
318	What did you do?<br><br>**[Interviewer: Do not read aloud - circle only ONE]**	Self-treated	1
		Went to friend/relative	2
		Went to a traditional healer	3
		Went to a TBA	4
		Went to a pharmacist	5
		Went to a private doctor	6 **skip to 320**
		Went to a health facility	7 **skip to 320**
		Other (specify) ________	96

319	What was (were) the reason(s) why you did not seek care from a doctor or health facility when you started having health problems?<br><br>**[Interviewer: Do not read aloud. Circle ALL that apply]**	Did not have money — A<br>Partner makes decisions, waited for apporval — B<br>Partner did not think I should go — C<br>Other family member's decision, waited for approval — D<br>Other family member did not think I should go — E<br>Disagreement among family members about what to do — F<br>Did not know where to go — G<br>Health facilities are too far — H<br>Afraid of mistreatment at facility — I<br>Afraid others would find out — J<br>Afraid of facility calling police — K<br>Lack (or difficulty) of transportation — L<br>Did not think health problem was serious — M<br>Did not have someone to take care of children — N<br>My religion does not allow me to go — O<br>Other (specify) — X<br>__________________________
320	How much time passed between the moment you realized you needed medical care and the time the decision was made that you needed to be taken to a health facility?<br><br>**[Interviewer: Please ask and indicate number of either minutes, hours, days, or weeks. If they cannot give a numerical answer, circle the column for unit of time it was within]**	Time from realized needed medical care to decision to go to facility (Time / This unit)<br>a. Minutes — 96<br>b. Hours — 96<br>c. Days — 96<br>d. Weeks — 96
321	During the time you waited between when your medical problems started and when you decided to go to a health facility, did your symptoms get worse?	Yes — 1<br>No — 2<br>Don't know — 98
322	Whose decision was it to seek medical care in a health facility?<br><br>**[Interviewer: Do not read aloud - circle only ONE]**	Respondent — 1<br>Husband/partner/boyfriend — 2<br>Couple jointly — 3<br>Mother/mother-in-law — 4<br>Some other relative — 5<br>Family decision jointly — 6<br>Neighbour/friend — 7<br>Someone else (specify) — 96<br><br>Don't know — 98
323	How much time passed between the moment when the decisions was made for you to be taken to a health facility and the time you actually got to the facility?<br><br>**[Interviewer: Please ask and indicate number of either minutes, hours, days, or weeks. If they cannot give a numerical answer, circle the column for unit of time it was within]**	Time from decision to facility (Time / This unit)<br>a. Minutes — 96<br>b. Hours — 96<br>c. Days — 96<br>d. Weeks — 96<br>**If respondent's answer is less than 24 hours, skip to 325**

324	What was (were) the reason(s) why you didn't go to a facility right away?<br><br>**[Interviewer: Do not read aloud. Circle ALL that apply]**	Lack of transport — A<br>Lack of money — B<br>Live too far from a health facility — C<br>Bad weather — D<br>No one to accompany respondent — E<br>No one to watch children — F<br>Indecision about which facility — G<br>Fear of other people knowing — H<br>Other (specify) — X<br><br>Don't know — Y
325	Did you come directly to this facility, or did you first go to another health facility before coming here?	Yes, came directly here — 1 → **skip to 329**<br>No, didn't come directly — 2<br>Don't know — 98
326	Where did you first seek care when you had the complication?<br><br>***[Interviewer: Circle only one]***	Central hospital — 1<br>Provincial hospital — 2<br>District hospital — 3<br>Mission hospital — 4<br>Rural hospital — 5<br>Primary health center — 6<br>Private hospital — 7<br>Private clinic — 8<br>NGO clinic — 9<br>Traditional healer — 10<br>Pharmacy — 11<br>Other — 98<br>(specify) ________
327	What was (were) the reason(s) you sought care there first?<br><br>**[Interviewer: Do not read aloud. Circle ALL that apply]**	Close to house — A<br>Charges low fees — B<br>Takes care of patients — C<br>Staff are very considerate — D<br>Privacy/small facility — E<br>I know someone who works at the facility — F<br>Other (specify) — X<br><br>Don't know — Y
328	Why did you leave the first facility for another facility?<br><br>**[Interviewer: Do not read aloud. Circle ALL that apply]**	Did not get better — A<br>Could not afford the charges — B<br>Was referred elsewhere — C<br>No staff on duty to provide treatment — D<br>No equipment to provide treatment — E<br>No bed was available — F<br>Too long of a wait/too many other patients — G<br>Treatment was unsatisfactory — H<br>Other (specify) — X<br><br>Don't know — Y
329	How much time passed between the time you arrived at <u>this</u> health facility and the time you were <u>first attended</u> to?<br><br>**[Interviewer: Please ask and indicate number of <u>either</u> minutes, hours, days, or weeks.If they cannot give a numerical answer, circle the column for unit of time it was within]**	See table below

Time from arrival at facility till attended to

	Time		This unit
a. Minutes			96
b. Hours			96
c. Days			96
d. Weeks			96

330	How much time passed between the time you were <u>attended to</u> and the time you received <u>complete treatment</u>?<br><br>**[Interviewer: Please ask and indicate number of <u>either</u> minutes, hours, days, or weeks.If they cannot give a numerical answer, circle the column for unit of time it was within]**	Time from attended to till complete treatment<br>(Time / This unit)<br>a. Minutes [ ][ ] 96<br>b. Hours [ ][ ] 96<br>c. Days [ ][ ] 96<br>d. Weeks [ ][ ] 96
331	Did you feel that the time that passed before you received treatment at this facility was reasonable, or did you feel that it was too long?	Reasonable 1 **skip to 333**<br>Too long 2
332	Why did it take time for you to be treated once you arrived at this facility?<br><br>**[Interviewer: Do not read aloud. Circle ALL that apply]**	I was treated immediately A<br>I did not have money at the time B<br>There was no bed for me C<br>No doctor/nurse was available D<br>There were many patients in line ahead of me E<br>Initally went to a wrong department F<br>Facility staff did not consider me a priority G<br>Would not treat me without my parent's consent H<br>Surgery room was busy I<br>No anesthesiologist was on duty J<br>Lack of supplies (i.e. blood, anesthesia) K<br>Problems with water/electricity L<br>Other (specify) X<br>Don't know Y
333	Please describe any of the following health problems you had <u>when you presented at this facility</u> as a result of this pregnancy complication as a mild, moderate or severe problem.<br><br>**[Interviewer: Read out each cateory, ie "Did you expereince bleeding?" If YES, ask if problem was mild, moderate, severe. Probe for any other problems]**	(see table below)
334	Thinking about all your health problems together, would you consider your complications to be mild, moderate or severe?	Mild 1<br>Moderate 2<br>Severe 3<br>Don't know 98
335	In spite of this/these health problem(s), were you able to do the following activities between the time the problem started, and when you decided to come to this facility to seek care?<br><br>**[Interviewer: Read each activity aloud and circle yes or no.]**	(see table below)
336	Did you or any other members of your household lose income because of your health problems between the time the problems started, and when you came to this facility for care?	Yes 1<br>No 2 → **skip to section 4**<br>Don't know 98
337	How much income did you, or your household, lose as a result of your health problems)not including cost of treatment or transportation)?	[ ][ ][ ][ ] USD $

333:

	Yes: Mild	Yes: Moderate	Yes: Severe	No
a. Bleeding	1	2	3	4
b. Pain	1	2	3	4
c. Injuries	1	2	3	4
d. Fever	1	2	3	4
e.Vaginal discharge (other than blood)	1	2	3	4
f. Other (specify) ______________	1	2	3	4

335:

	Yes	No	N/A
a. Cooking	1	2	99
b. Housework (other than cooking)	1	2	99
c. Going to work	1	2	99
d. Caring for other family members	1	2	99

Section IV: INDUCED ABORTION

In the next few questions, I want to ask you about the pregnancy that led to the treatment you are receiving in this facility. They are quite sensitive. I would like to ask you about whether your physical condition has resulted from a spontaneous or an induced abortion. I want to reassure you that, as I said before, this information is highly confidential, and we will protect your identity. Your honest answers to these questions will help us understand problems women face and how the country can better respond to them. I will now ask you questions about induced abortions and unintended pregnancy.

No.	Question	Response	Code	Skip
401	Do you know anyone who had an induced abortion ?	Yes	1	
		No	2	**→ skip to 403**
402	Who do you know who had an induced abortion? **[Interviewer: Explain to the patient that we are not interested in the name of the person but his/her relationship to the patient. Circle all that apply.]**	Friend	A	
		Neighbor	B	
		Sister/Sister-in-law	C	
		Mother/Mother-in-law	D	
		Other relative (specify) ______	E	
		Friend	F	
		Other (specify) ______	X	
403	Did you or someone else do anything to interfere with the continuation of this pregnancy?	Yes	1	
		No	2	**→Skip to concluding statement**
404	What was the reason that led you to make the decision not to continue with the pregnancy? **[Interviewer: Do not read aloud. Circle all that the respondent says.]**	Husband/partner did not want anymore children	A	
		Have too many children	B	
		Cannot afford anymore children	C	
		Too soon after last pregnancy	D	
		Would have to drop out of school	E	
		Would have to leave job	F	
		Too young	G	
		Too old	H	
		Not married	I	
		The pregnancy was not with husband/partner	J	
		The pregnancy was the result of rape/incest	K	
		It endangered my health	L	
		The foetus would have been handicapped/foetal anomaly	M	
		I am HIV positive	N	
		Other (specify) ______	X	

405 How much time passed between the moment you realized you were pregnant, and when you decided you wanted to terminate the pregnancy?

[Interviewer: Please ask and indicate number of <u>either</u> minutes, hours, days, or weeks. If they cannot give a numerical answer, circle the column for unit of time it was within]

Time from reallizing pregnant to decision to terminate	Time		This unit
a. Minutes			96
b. Hours			96
c. Days			96
d. Weeks			96

406	Did you discuss the possibility of stopping this pregnancy with anyone?	Yes 1<br>No 2 → **skip to 412**
407	With whom did you discuss the possibility of stopping this pregnancy?<br><br>**[Interviewer: Do not read aloud. Circle all that apply. Probe, "Anyone else?" until complete.]**	Husband/partner A<br>Mother B<br>Sister C<br>Mother-in-law D<br>Other relative (specify) E<br>Friend F<br>Other (specify) X
408	Did any of these people think that you should stop the pregnancy?	Yes 1<br>No 2 → **skip to 410**
409	Which of these people thought you should stop the pregnancy?<br><br>**[Interviewer: Read each of the options circled from 407 aloud. Circle all that apply.]**	Husband/partner A<br>Mother B<br>Sister C<br>Mother-in-law D<br>Other relative (specify) E<br>Friend F<br>Other (specify) X
410	Who made the decision to stop this pregnancy?<br><br>**[Interviewer: Do not read aloud. If respondent says that she made the decision herself, ask her if anyone else helped her make that decision and circle that person in the list. Circle ALL that apply.]**	Woman alone A<br>Husband/partner B<br>The couple together C<br>Woman's parents D<br>Husband/partner's parents E<br>Other family member F<br>Other (specify) X
411	How many weeks or months pregnant were you when you first tried to stop this pregnancy?	a. Weeks ☐☐<br>***OR***<br>b. Months ☐☐<br>Unknown gestation 98
412	Women sometimes take more than one attempt to stop a pregnancy. Did you attempt to stop this pregnancy more than once?	Yes 1<br>No 2 → **skip to 415a**

413	What did you first do to end this pregnancy?<br><br>**[Interviewer: Do not read aloud. Circle only ONE]**	Drank herbal concoction 1<br>Drank other home remedies 2<br>Used any herbal enema 3<br>Inserted herb/object/other substance into vagina 4<br>Heavy Massage 5<br>D&C 6<br>Manual vacuum aspiration 7<br>Injection 8<br>Saline instillation 9<br>Misoprostol/cytotec 10<br>Oxytocin 11<br>Other tablets 12<br>Catheter 13<br>Excessive physical activity 14<br>Don't know/can't remember 98<br>Other (specify) 96<br>________________
414	How much time passed between your first attempt to end this pregnancy and when you actually succeeded in stopping it?<br><br>**[Interviewer: Please ask and indicate number of either minutes, hours, days, or weeks. If they cannot give a numerical answer, circle the column for unit of time it was within]**	Time from first attempt to end pregnancy and when successfully stopped it<br>(Time / This unit)<br>a. Minutes __ __ 96<br>b. Hours __ __ 96<br>c. Days __ __ 96<br>d. Weeks __ __ 96
415<br><br><br>415a	What was the last thing you did to try to end this pregnancy?<br><br>**[Interviewer: Do not read aloud. Circle only ONE]**<br><br>What did you do to end this pregnancy?	Drank herbal concoction 1<br>Drank other home remedies 2<br>Used any herbal enema 3<br>Inserted herb/object/other substance into vagina 4<br>Heavy Massage 5<br>D&C 6<br>Manual vacuum aspiration 7<br>Injection 8<br>Saline instillation 9<br>(1–9: Skip to 417)<br>Misoprostol/cytotec 10<br>Oxytocin 11<br>Other tablets 12<br>Catheter 13<br>Excessive physical activity 14<br>Don't know/can't remember 98<br>Other (specify) 96<br>(11–96: Skip to 417)<br>________________
416	Where did you acquire misoprostol?<br><br>**[Interviewer: Circle all the apply. After completing, skip to 418]**	Doctor's private office A<br>Nurses' or clinical officers' private office B<br>Health facility (public) C<br>Health facility (private) D<br>NGO E<br>Traditional healer F<br>Pharmacy G<br>Ordered online H<br>Markets or street vendor I<br>Other (specify) X<br>________________

417	Who provided this method that was finally used to stop the pregnancy?<br><br>**[Interviewer: Explain to the patient that we are not interested in the name of the person but his/her position/cadre. This could be a doctor, nurse, relative, or the woman herself.]**<br><br>**[Interviewer: Circle all that apply]**	Doctor A<br>Nurse B<br>Trained Midwife C<br>Traditional healer/TBA D<br>Pharmacist E<br>Self -induced F<br>Parent G<br>Relative/ Friend H<br>Other (specify) X<br>________________
418	Who paid your expenses for trying to stop this pregnancy?<br><br>**[Interviewer: Do not read aloud. Circle all that apply.]**	Woman herself A<br>Husband/partner B<br>Parent C<br>Other family member D<br>Friend E<br>Other (specify) X<br>________________
419	How much did it cost to stop this pregnancy?	☐☐☐☐ USD $<br>98 Don't know

CONCLUDING STATEMENT

This is the end of my questions, and I want to thank you for your help on this important project. If you have any questions for me, or concerns, please feel free to raise them now. I would be happy to discuss them with you. If you felt uncomfortable or emotionally disturbed in the course of answering some questions, and would like a referral to counseling services, I can provide you with a list of referral services nearby. Would you like me to provide you with this list?

To enable us have a better understanding of the issues relating to pregnancies that did not result in a live birth, we would like to request your permission to ask your doctor or other hospital staff more questions about your condition when you came to the hospital, your symptoms, treatment, and other services you received here. I am now going to read to you a consent form asking for your consent to interview your provider about your condition.

[Interviewer: Begin administering consent form to contact provider. Remember to fill in the interview outcome in 010 on the cover page of the questionnaire.]

012 **END TIME** ☐☐ hr ☐☐ min

COMMENTS: ______________________________

Prospective Morbidity Survey Process Completion Checklist

Please verify that each task below has been completed. If you are responsible for the task, add your signature to the corresponding task and date when the activity was completed.

	Field level				Office level	
**Questionnaire checked for:**	Interviewer	Date	Supervisor	Date	Data entry clerk signature	Date
**a. Questionnaire completeness checked**						
**b. Clear handwriting checked**						
**c. Appropriate response type checked**						
**d. Skip patterns properly followed**						

I verify that all of this information is completed.
Supervisor's signature: ______________________

FIRST DATA ENTRY COMPLETE: ______________________

SECOND DATA ENTRY COMPLETE: ______________________

PROSPECTIVE MORBIDITY SURVEY IN ZIMBABWE 2016

Questionnaire for Provider

University of Zimbabwe-University of California San Francisco, Zimbabwe Ministry of Health and Child Care, and Guttmacher Institute

Questionnaire ID: ☐☐☐☐☐☐☐☐
(number should be same as patient questionnaire)

BACKGROUND INFORMATION		CODES
**Instructions: Please fill out 001-005 before starting the interview with the provider, and 006 and 007 upon completion.**		
001	Health Facility Name ______________________	
002	Health Facility Code	☐☐☐☐
003	Date of Admission	☐☐ ☐☐ ☐☐ (DD MM YY)
004	Date of Discharge	☐☐ ☐☐ ☐☐ (DD MM YY)
005	Date Patient was Interviewed	☐☐ ☐☐ ☐☐ (DD MM YY)
006	Outcome of interview:	1 Interview was completed<br>2 Patient did not consent to interview<br>3 Patient did not consent to provider interview<br>4 Patient could not consent to interview<br>5 Patient died during care<br>6 Other - please specify ______________________
007	Patient was:	1 Near-miss<br>2 Not near-miss<br>The WHO defines "near miss" as a woman who nearly died but survived a complication that occurred during pregnancy or within 42 days of termination of pregnancy.

Section I : CLINICAL INFORMATION (General information, vital signs, vaginal examination, physical examination)

Instructions: Please fill out Section I in consultation with the provider and the patient's chart.

GENERAL INFORMATION

101	Patient was:	1 Day patient<br>2 In-patient: number of nights ☐☐
102	Presenting complaints:<br>**[Interviewer: Circle all that apply]**	A Bleeding, blood loss<br>B Fever No. of days of fever before admission (if known) ☐☐<br>C Pain<br>D Other – specify: ______________ ☐☐
103	Estimated gestational age:	☐☐ Weeks from last menstrual period<br>Unknown LMP 96 → Weeks estimated ☐☐

VITAL SIGNS

Please record the following vital signs of the client on her arrival for seeking care, using the patient's chart.

104	A. Body temperature (rounded to the tenth decimal point)	☐☐ • ☐ ° C
	B. Pulse rate	☐☐☐ per minute
	C. Systolic blood pressure	☐☐☐ mmHg
	D. Diastolic blood pressure	☐☐☐ mmHg

	**VAGINAL EXAMINATION FINDINGS ON ARRIVAL TO THE FACILITY FOR THE CURRENT CARE:**	
105	Evidence of foreign body in vagina, cervix, or uterus	1 Yes<br>2 No
106	On examination, which of the following mechanical injuries did you detect?<br><br>**[Interviewer: Circle all that apply.]**	A Cervical laceration<br>B Cervical tears<br>C Tenaculum bites of the cervix<br>D Mechanical injury of uterus<br>E Intra-abdominal injury<br>F Puncture marks on cervix or vagina<br>X No sign of mechanical injury
107	On vaginal examination, did you note offensive or foul smelling products of conceptus?	1 Yes<br>2 No
	**PHYSICAL EXAMINATION FINDINGS ON ARRIVAL TO THE FACILITY FOR THE CURRENT CARE:**	
108	Did you find any sign of infection on examination?	1 Yes<br>2 No
109	What complications did you find on examination and investigation?<br>**[Interviewer: Circle all that apply.]**	A Abdominal/uterine tenderness<br>B Pelvic abscess<br>C Pelvic peritonitis<br>D Generalized peritonitis<br>E Uterine perforation<br>F Sepsis/septicemia<br>G Hemorrhagic shock<br>H Hemorrhage<br>I Septic shock<br>J Purulent discharge<br>K Anemia<br>L Fever<br>X Other: ____________<br>____________<br>Z None
110	Did you note any sign of organ/system failure on examination?	1 Yes<br>2 No ➔**Skip to 201**
111	What signs of organ/system failure did you find on examination and investigation?<br><br>**[Interviewer: Circle all that apply.]**	A Respiratory distress syndrome<br>B Renal failure<br>C Liver failure<br>D Cardiac failure<br>E Coma<br>F Coagulation defect (DIC)<br>X Others (specify) ____________

Section II: DIAGNOSIS ON ARRIVAL TO THE FACILITY FOR CURRENT CARE

No.	Question	Response	Code	Skip
201	Based on your overall assessment of the client and your clinical examination findings, how would you classify the patient's aboriton? **[Interviewer: Read options and circle the one answer respondent gives.]**	Certaintly induced	1	
		Probably induced	2	
		Possibly induced	3	
		Most likely spontanous	4	
		Don't know	96	
202	The diagnosis is: **[Circle only one response.]**	Inevitable abortion	1	
		Incomplete abortion	2	
		Missed abortion	3	
		Complete abortion	4	**skip to 304**
		Other: (specify) ________	96	
203	Is the abortion also septic?	Yes	1	
		No	2	

Section III : TREATMENT

No.	Question	Response	Code	Skip
301	Describe the main procedure that was used in the management of the patient's condition. **[Circle only one response.]** **[Only select option 4 (medical abortion or misoprostol) or option 7 (oxytocin) if it was provided as the only procedure given to the patient and not given alongside other treatment.]**	Dilation and curettage (D&C)	1	
		Manual vacuum aspiration(MVA)	2	
		Electric vacuum aspiration(EVA)	3	
		Medical abortion (e.g. misoprostol/Cytotec)	4	
		Digital Evacuation	5	
		Forceps Evacuation	6	
		Oxytocin	7	
		Evacuation by curettage	8	
		Other (specify) ________	96	
302	Where was the evacuation procedure performed?	Operating theatre	1	
		General outpatient clinic	2	
		Outpatient MCH clinic	3	
		Outpatient gynaecological clinic	4	
		General surgical ward	5	
		Gynaecological/Obstetric ward	6	
		PAC or Emergency Obstetric Unit	7	
		Female ward	8	
		Intensive care unit (ICU)	9	
		Emergency ward	10	
		Other ward (specify) ________	96	
303	The procedure was performed primarily by: **[Interviewer: Circle only one response.]**	Ob/Gyn	1	
		Medical Officer/ GP	2	
		Nurse	3	
		Midwife	4	
		Clinical Officer	5	
		Other (specify) ________	96	
304	Was the client provided any pain medication during the evacuation procedure?	Yes	1	
		No	2	**➔Skip to 306**

No.	Question	Response	Code	Skip
305	What did the client receive? **[Interviewer: Circle all that apply.]**	General anesthesia	A	
		Para cervical LA/ Para Cervical block	B	
		Sedation	C	
		Analgesics	D	
		Other	X	
		(specify) ______		
306	Was the client administered antibiotics during her current visit at this facility?	Yes	1	
		No	2	➔**Skip to 309**
307	Type of antibiotics given:	Oral only	1	
		IV only	2	
		Combined (oral + IV)	3	
308	Who supplied the antibiotics, the facility or the client?	Facility supplied	1	
		Client supplied herself	2	
		Both	3	

No.	Question		Y	N	Item
309	Was the client given the following drugs or products during her current visit at this facility? **[Interviewer: Please read out each category, and write a response.]**	A	1	2	Intravenous fluids
		B	1	2	Blood or blood products
		C	1	2	Oxytocics (oxytocin or ergometrine)
		D	1	2	Misoprostol

Section IV: SURGICAL PROCEDURES FOR COMPLICATIONS

No.	Question	Response	Code
401	What surgical procedures were performed on the client? **[Interviewer: Circle all that apply]**	Hysterectomy	A
		Salpingectomy	B
		Abscess drainage	C
		Repair of cervical tear	D
		Repair of vaginal tears	E
		Repair of vulva/perineal tears	F
		Repair of perforated uterus	G
		Repair of gut perforation	H
		Laparatomy	I
		Other	X
		(specify) ______	
		None	Y

Section V: POSTABORTION CONTRACEPTION

No.	Question	Response	Code	Skip
501	Was the client counseled on contraception upon discharge?	Yes	1	
		No	2	
		Not discharged yet	3	
		Don't know	98	
502	Was the client given modern contraception?	Yes	1	➔**Skip to 504**
		No	2	
		Don't know	98	➔**Skip to 601**
503	Was the client referred elsewhere for modern contraception?	Yes	1	➔**Skip to 601**
		No	2	➔**Skip to 601**

504	Which of the following methods did you offer to the client or her partner?<br><br>**[Interviewer: Ask whether respondent offered any of the methods listed and circle all that apply. Then probe by asking:]**<br><br>Did you offer them any other methods?	Male condom A<br>Female condom B<br>Pills C<br>Injectables D<br>Implants E<br>Intrauterine devices (IUD/IUCD) F<br>Female sterilization G<br>Vasectomy H<br>Other X<br>(specify) ____________
505	What contraceptive method or methods did the client or her partner receive?<br><br>**[Interviewer: Circle all that apply.]**	Male condom A<br>Female condom B<br>Pills C<br>Injectables D<br>Implants E<br>Intrauterine devices (IUD/IUCD) F<br>Female sterilization G<br>Vasectomy H<br>Other X<br>(specify) ____________<br>Not yet discharged Y<br>Don't know Z
**Section VI: OUTCOME OF CLINICAL MANAGEMENT OF CURRENT CARE**		
601	The outcome of current care was:<br><br>**[Interviewer: Comment could include the cause of leaving against medical advice or the condition of the woman when she left.]**	Discharged well 1<br>Died 2<br>Still hospitalized 3<br>Left against medical advice 4<br>(comment) ____________<br>Referred to another facility 5<br>(specify) ____________
602	How long did the client stay in the facility for care?	Less than 12 hours 1<br>12 to 24 hrs 2<br>More than 24 hours 3
**CONCLUDING STATEMENT**		
**This is the end of my questions, and I want to thank you for your help on this important project.**		

Commentary: ____

DATA QUALITY CHECK

Please verify that each task below has been completed. If you are responsible for the task, add your code to the corresponding task and date when the activity was completed.

	Field level				Office level	
**Questionnaire checked for:**	Interviewer signature	Date	Supervisor signature	Date	Data entry clerk signature	Date
**a. Completeness**						
**b. Clear handwriting**						
**c. Appropriate response type**						
**d. Skip patterns properly followed**						

I verify that all of this information is completed.
Supervisor's signature: ____

FIRST DATA ENTRY COMPLETE: ____

SECOND DATA ENTRY COMPLETE: ____
