# Supplementary material for: Abortion in Zimbabwe: A national study of the incidence of induced abortion, unintended pregnancy and post-abortion care in 2016
Source: PLoS One. 2018 Oct 24;13(10):e0205239. doi: 10.1371/journal.pone.0205239 (PMC6200425; doi:10.1371/journal.pone.0205239)
Supplement: S3 File — (PDF) [file pone.0205239.s003.pdf]

STUDY OF ABORTION AND WOMEN'S HEALTH IN ZIMBABWE

UZ UCSF
Saving lives through innovative research strategies

GUTTMACHER INSTITUTE

HEALTH PROFESSIONALS SURVEY

IDENTIFICATION

M1 **RESPONDENT IDENTIFICATION NUMBER** ________ [ ][ ][ ]

M2 **PROVINCE**

A. Bulawayo
B. Harare
C. Manicaland
D. Mashonaland Central
E. Mashonaland East
F. Mashonaland West
G. Masvingo
H. Matabeleland North
I. Matabeleland South
J. Midlands

M3 **DISTRICT** ________ [ ][ ]

M5 **DATE OF INTERVIEW** [ ][ ] day [ ][ ] month [2][0][1][6] Year

M6 **INTERVIEWER:** [ ][ ]

M7 **TIME STARTED:** [ ][ ] h [ ][ ] m

M8

INTERVIEW OUTCOME (1ST)	INTERVIEW OUTCOME (2ND)	INTERVIEW OUTCOME (3RD)
1 Completed	1 Completed	1 Completed
2 Incomplete	2 Incomplete	2 Incomplete
3 Refused	3 Refused	3 Refused
4 Not available for interview	4 Not available for interview	4 Not available for interview

Section 1: Basic Information

No.	Questions and Filters	Responses and Codes (Please circle neatly)	
101	Gender of the respondent<br>**[Interviewer: circle the category that applies to the respondent. Do not ask this question aloud.]**	1	Male
		2	Female
102	What is your primary profession?<br><br>**[Interviewer: Do not read. Circle only one response. If respondent gives more than one profession, select the one that accounts for the greatest proportion of the respondent's time.]**	1	General physician
		2	Obstetrician/Gynecologist
		3	Nurse/Midwife
		4	Community health worker
		5	Community-based distributor (CBD)
		6	Village health worker
		7	Traditional healer
		8	Other health professional (*specify*): ___________________
		9	Program manager/health administrator
		10	Policy maker/policy advisor
		11	Activist/lawyer (e.g. in women's organization/issues)
		12	District Social Welfare Officer
		13	Village head/chief
		14	Community leader / youth leader
		15	Journalist / media person
		16	Researcher
		17	Lecturer/teacher
		96	Other *(specify):* ________________________________
103	In which sector do you work primarily?<br><br>**[Interviewer: If the respondent works in more than one sector, circle the category corresponding to the sector where s/he contributes the most time.]**	1	Private sector
		2	Public sector (including government)
		3	Non-governmental organization
		96	Other *(specify)* ___________________________
104	How long have you been working in your current profession? **[In completed years]**	☐☐	Years
105	In what year were you born? **[Insert last two digits of the calendar year, unless they were born after 2000]**	☐☐	

No.	Questions and Filters		Province	Urban area = 1<br>Rural area = 2<br>Both = 3
106	In which Province of the country have you worked during the last 5 years? Did you work in urban areas or rural areas or both?<br><br>1= urban, 2 = rural, 3 = both<br><br>**[Only fill in for provinces where respondent has worked. Provinces where respondent has not worked should be left blank.]**	A	**Bulawayo**	
		B	**Harare**	
		C	**Manicaland**	
		D	**Mashonaland Central**	
		E	**Mashonaland East**	
		F	**Mashonaland West**	
		G	**Masvingo**	
		H	**Matabeleland North**	
		I	**Matabeleland South**	
		J	**Midlands**	

107	Please identify the different contexts in which you have encountered the issue of abortion as part of your professional experience?<br><br>**[Interviewer: Do not read. Please circle all that apply.]**	A<br>B<br>C<br>D<br>E<br>F	In a public health facility framework<br>In a private clinic framework<br>Non-medical framework (research, policy-making, counseling, law) (Specify) __________________________<br>Through colleagues/professional networks<br>Through personal networks<br>Other *(specify)* : ____________________________<br>____________________________
108	What is the highest level of education you have completed?	1<br>2<br>3<br>4<br>5<br>6<br>7<br>8	No education<br>Did not complete primary<br>Completed primary<br>Some secondary<br>O-levels<br>A-levels<br>University or college<br>Post-graduate education

Section 2: Service provision

In Zimbabwe, very little is known about the provision of induced abortion—that is, steps taken or procedures done to end a pregnancy. Induced abortion may be done at any gestation, and it may be done by both trained and untrained providers in safe and unsafe conditions.

We would like to have your opinion about several aspects of this area of reproductive behavior, to the extent that you are able to give an informed professional opinion. In this questionnaire, by abortion, we will be referring to induced abortion only.

201 First, I would like to learn about the methods currently used by Zimbabwean women to induce an abortion, regardless of the type of practitioner who may use the method or whether it takes place in a facility. As far as you are aware, is [type of method] used to induce an abortion?

[Interviewer: make your way down the list of methods, asking about induced abortions in Zimbabwe.]

**Type of Method [Read each method type]**	**No**	**Yes**	**DK**
**a. Electric or manual vacuum aspiration (MVA)**	0	1	8
**b. Uterine evacuation with sharp curette (D&E or D&C)**	0	1	8
**c. Oral introduction of drugs, solutions or other substances (i.e. through the mouth)**			
c1. Misoprostol (Cytotec) alone, taken orally	0	1	8
c2. Decoction, drink, tea, local herbs	0	1	8
c3. Overdose of pharmaceuticals (e.g. aspirin, chloroquine, metronidazole)	0	1	8
c9. Other oral methods *(specify)* ______________________________	0	1	8
**d. Vaginal introduction of drugs, solutions or other materials**			
d1. Misoprostol (Cytotec) taken vaginally	0	1	8
d2. Herbs or solutions (using any form of insertion into the vagina)	0	1	8
d3. Foreign objects (e.g. sticks, metal objects)	0	1	8
d4. Caustic agent (e.g. washing powder)	0	1	8
d5. Catheter (with or without solution)	0	1	8
d9. Other vaginal methods *(specify)* ______________________________	0	1	8
**e. Other means** (*specify any additional method(s) not listed above):*			
______________________________		1	
______________________________		1	
______________________________		1	
		1	

Misoprostol questions

[Interviewer: If the respondent answered "No" to c1 AND d1 in question 201, skip to question 204]

You mentioned earlier that misoprostol (or Cytotec) is sometimes being used in Zimbabwe for induced abortion. I would like to ask your perceptions about how misoprostol is being used for induced abortion in Zimbabwe.

202 In what year do you think misoprostol first started being used in Zimbabwe to induce abortion?

[ ][ ][ ][ ]

8 Don't know

203 Now I want to ask you about the main types of **places** people could go to get misoprostol to induce an abortion in Zimbabwe. Indicate whether, in your opinion, women in Zimbabwe access misoprostol from these places commonly, sometimes, or rarely/never.

[Interviewer: Read each place listed below and circle the the appropriate response]

**Type of Provider**	Commonly	Sometimes	Rarely/ never	Don't know
A. Doctor's private office	1	2	3	8
B. Nurse's or clinical officers' private office	1	2	3	8
C. Health facilities (public)	1	2	3	8
D. Health facilities (private)	1	2	3	8
E. NGOs	1	2	3	8
F. Traditional healers	1	2	3	8
G. Pharmacy	1	2	3	8
H. Markets or street vendors	1	2	3	8
X. Other (specify) ______________________________	1	2	3	8

All Methods

Now I'd like to talk to you about all kinds of abortion.

In your opinion, what is the most common method used to induce abortion by the following practitioners?

By misoprostol, we mean an oral or vaginal introduction of drugs misoprostol. By other types of abortion, we mean oral introduction of other substances, vaginal introduction of solutions, or other materials, phsyical methods, or any other means.

[Interviewer: Read out the main types of methods, and circle only one response for each provider type.]

		a. Surgical: Electric or manual vacuum aspiration (MVA)	b. Surgical: Uterine evacuation with sharp curette (D&E or D&C)	c. Misoprostol	d. Other methods of abortion	f. Don't know
204	Medical doctors?	1	2	3	4	8
205	Nurses, midwives or other trained providers?	1	2	3	4	8
206	Traditional provider (anyone without formal training)?	1	2	3	4	8
207	Pharmacists?	1	2	3	4	8
208	Women to self-induce abortion?	1	2	3	4	8

For many of the following questions, I want you to consider two income groups – the poor and the relatively well-off (non-poor). Each question will be asked separately about poor and non-poor women who live in Zimbabwe.

[Interviewer: You can mention that there are not exact definitions for "poor" and "non-poor," but by "poor" we mean women with lower income levels.]

209-210 I will mention the main types of people who perform induced abortions in Zimbabwe. These includes doctors, nurses/midwives, tradiatinoal providers, pharmcists, and women who self-induce.

Considering first **poor women**, indicate whether, in your opinion, women who seek abortions go to the following types of providers commonly, sometimes or rarely/never.

[Interviewer: Read each type of provider and circle the respondent's answers for poor urban women in the first 3 columns. Then ask the next question.]

Now indicate whether, in your opinion, **non-poor women** who seek abortions go to the following types of providers commonly, sometimes or rarely/never.

**Type of Provider** **[Read each type]**	**C=Commonly** **S=Sometimes** **R=Rarely/Never**	**209 Poor Women**			**210 Non-Poor Women**		
		**C**	**S**	**R**	**C**	**S**	**R**
a. Doctor		1	2	3	1	2	3
b. Nurse/midwife or other trained provider (not a doctor)		1	2	3	1	2	3
c. Traditional provider (anyone without formal training)		1	2	3	1	2	3
d. Pharmacist		1	2	3	1	2	3
e. Woman herself - self-induced		1	2	3	1	2	3
x. Other *(specify):* ______________________________		1	2	3	1	2	3

211 In your opinion, how much do women living in Zimbabwe pay for abortions from each of the providers we just discussed? It can be difficult to estimate because costs can vary widely. Can you provide what you think is the **minimum** and **maximum** paid by women in Zimbabwe? I will read our each type of provider.

[Interviewer : Only ask this question about providers that are used commonly or used sometimes (Q.209 =1 or 2 OR Q.210 = 1 or 2). The cost should include the cost of services without transportation If DK, put a slash across the minimum and maximum boxes.]

**Type of Provider**	**Amount paid: 1. Minimum**	**Amount paid: 2. Maximum**
a. Doctor		
b. Nurse/midwife or other trained provider (not a doctor)		
c. Traditional provider (anyone without formal training)		
d. Pharmacist		
e. Woman herself - self-induced		
x. Other **(see 209-210)**		

URBAN AREAS

Now I am going to ask you questions about how women in urban areas of Zimbabwe obtain induced abortions. After I finish, I will ask you the same questions about women in rural areas. Please only answer about urban women until I mention we are switching to questions on rural women.

We want to understand what methods both poor and non-poor women in **urban** areas in Zimbabwe use to obtain an induced abortion. We want you to consider three different types of abortions a woman may have: surgical abortion, abortion through the use of misoprostol, and all other types. By surgical abortions, we mean vacuum aspiration (MVA or EVA), dilation and curettage (D&C), or a catheter abortion in a surgical abortion. Each question will be asked separately about poor and non-poor women who live in urban areas.

212-213

I am going to ask you what percentage of poor women in **urban** areas go to each type of provider. The sum of women in each of the 3 categories should add up to 100%.

First, what percentage of all induced abortions among **poor women in urban areas** do you think are done with **surgical abortion**?

[Before asking about misoprostol, check back to Q201 options c1 and d1. If respondent answered No, fill in 0] What percentage of all induced abortions among poor women in urban areas do you think are done with **misoprostol**?

What percentage of all induced abortions among poor women in urban areas do you think are **other types of abortion**?

[Interviewer: Please ensure all percentages total to 100%. If not, please ask respondent to adjust percentages. If the responsent says that no women to any of the categories, fill in "00".
After asking about poor women in urban areas, go back through and repeat questions for non-poor women in urban areas.]

	a. Surgical abortion	b. Misoprostol	c. Other types of abortion	**Total**
Q212 Urban poor women				**100%**
Q213. Urban non-poor women				**100%**

214-216

Among poor urban women who have each type of abortion, I now want to ask you what percentage of them go to each type of provider we discussed earlier.

First, what percentage of all induced abortions performed through **surgical abortion** to **poor** women in **urban** areas do you think are being performed by doctors or by nurses, midwives and other trained proivders? Give an appproximate percentage keeping in mind that all providers must sum to 100%.

[**Interviewer: only ask this following question about misoprostol if respondent said answered greater than "00"% about misoprostol in Q212b]:**
What percentage of all induced abortions performed through **misoprostol** to **poor** women in **urban** areas do you think are being performed by each type of provider?

If the misoprostol has been prescribed by a doctor, the doctor is the provider. If the woman gets the misoprostol from a pharmacist without a prescription, the pharmacist is the provider. Self-inducing using misoprostol includes them accessing misoprostol on the black market or from other sources other than the providers.

Next, what percent of all induced abortions through **all other types of abortion** to **poor** women in **urban** areas do you think are being performed by each type of provider?

**Type of provider**	% of poor urban women going to each type of provider for		
	Q214. **Surgical abortion**	Q215. **Misoprostol**	Q216. **Other types of abortion**
a. Doctor			
b. Nurse/midwife or other trained provider			
c. Traditional provider (anyone without formal training)			
d. Pharmacist			
e. Woman (self-induced)			
**Total**	**100%**	**100%**	**100%**

217-219

Now I am going to ask you the same questions as before, but now about non-poor women in urban areas.

What percentage of all induced abortions performed through **surgical abortion** to **non-poor** women in **urban** areas do you think are being performed by each type of provider?

[Interviewer: only ask this following question about misoprostol if respondent said answered greater than "00"% about misoprostol in Q213b]: What percent of all induced abortions performed through **misoprostol** to **non-poor** women in **urban** areas do you think are being performed by each type of provider?

Next, what percent of all induced abortions through **all other types of abortion** to **non-poor** women in **urban** areas do you think are being performed by each type of provider? Give an appproximate percentage keeping in mind that all providers must sum to 100%.

**Type of provider**	% of non-poor urban women going to each type of provider for		
	Q217. **Surgical abortion**	Q218. **Misoprostol**	Q219. **Other types of abortion**
a. Doctor			
b. Nurse/midwife or other trained provider			
c. Traditional provider (anyone without formal training)			
d. Pharmacist			
e. Woman (self-induced)			
**Total**	**100%**	**100%**	**100%**

RURAL AREAS

Now I am going to ask you the same questions as above, but this time for rural areas.

Now we want to understand what methods both poor and non-poor women in **rural** areas in Zimbabwe use to obtain an induced abortion. When we were talking about urban areas, we were talking about three methods: surgical, medical, and all other types of abortion. Now when we talk about rural areas, I'd like to talk about the same three methods. Again, each question will be asked separately about poor and non-poor women who live in rural areas.

220-221

I am going to ask you what percentage of poor women in **rural** areas go to each type of provider. The sum of women in each of the 3 categories should add up to 100%.

What percentage of all induced abortions among **poor women in rural areas** do you think are done with **surgical abortion**?

[Before asking about misoprostol, check back to Q201 options c1 and d1. If respondent answered No, fill in 0] What percentage of all induced abortions among poor women in rural areas do you think are done with **misoprostol**?

What percentage of all induced abortions among poor women in rural areas do you think are **other types of abortion**?

[Interviewer: Please ensure all percentages total to 100%. If not, please ask respondent to adjust percentages. If the responsent says that no women to any of the categories, fill in "00". After asking about poor women in rural areas, go back through and repeat questions for non-poor women in rural areas.]

	a.Surgical abortion	b.Misoprostol	c. Other types of abortion	**Total**
Q220. Rural poor women				**100%**
Q221. Rural nonpoor women				**100%**

222-224 Among poor rural women who have each type of abortion, I want to know what percentage of them go to each type of provider we discussed earlier.

What percent of all induced abortions performed through **surgical abortion** to **poor** women in **rural** areas do you think are being performed by each type of provider? Give an appproximate percentage keeping in mind that all providers must sum to 100%.

[**Interviewer: only ask this following question about misoprostol if respondent said answered greater than "00"% about misoprostol in Q220b]:** What percent of all induced abortions performed through **misoprostol** to **poor** women in **rural** areas do you think are being performed by each type of provider?

Next, what percent of all induced abortions through **all other types of abortion** to **poor** women in **rural** areas do you think are being performed by each type of provider?

Type of provider	% of poor rural women going to each type of provider for		
	Q222. **Surgical abortion**	Q223. **Misoprostol**	Q224. **Other types of abortion**
a. Doctor			
b. Nurse/midwife or other trained provider			
c. Traditional provider (anyone without formal training)			
d. Pharmacist			
e. Woman (self-induced)			
**Total**	**100%**	**100%**	**100%**

225-227 Now I want to know the answers to the same questions I just asked, but about non-poor rural women.

What percent of all induced abortions performed through **surgical abortion** to **non-poor** women in **rural** areas do you think are being performed by each type of provider?

[**Interviewer: only ask this following question about misoprostol if respondent said answered greater than "00"% about misoprostol in Q221b] :**What percent of all induced abortions performed through **misoprostol** to **non-poor** women in **rural** areas do you think are being performed by each type of provider?

Next, what percent of all induced abortions through **all other types of abortion** to **non-poor** women in **rural** areas do you think are being performed by each type of provider?

Type of provider	% of non-poor rural women going to each type of provider for		
	Q225. **Surgical abortion**	Q226. **Misoprostol**	Q227. **Other types of abortion**
a. Doctor			
b. Nurse/midwife or other trained provider			
c. Traditional provider (anyone without formal training)			
d. Pharmacist			
e. Woman (self-induced)			
**Total**	**100%**	**100%**	**100%**

URBAN AND RURAL AREAS

Now I want to ask you about rural and urban women together. We are aware that Zimbabwean women frequently seek medical care outside of the country, including to have an induced abortion.

228	Out of 100 women living in this **province** who have an induced abortion, how many do you think obtain their abortion outside of the country?	___ women
229	a. Women may also buy or import drugs from South Africa to induce an abortion in Zimbabwe. Have you heard of women importing misoprostol from South Africa?	a. Misoprostol: 1 Yes; 2 No; 8 Don't know
	b. Have you heard of women importing mifepristone, a drug that is often used in combination with misoprostol to induce abortion?	b. Mifepristone: 1 Yes; 2 No; 8 Don't know

Section 3: Abortion Complications

Now, I will talk to you about abortion complications from induced abortion, meaning an abortion that is provoked. As you know, complications that result from induced abortion range in severity. When we speak of abortion complications, we are referring to those consequences **that are severe enough to need treatment** in a health facility**, whether or not the woman actually obtains health care.**

Complications due to abortion, as defined here, include not only the extremely serious cases such as those with sepsis or a perforated uterus, but also those cases which are termed "incomplete abortions," which are usually identified by heavy bleeding, and which present a somewhat less severe health risk to the woman, but which, nevertheless, require treatment in a health facility. In answering the following set of questions concerning abortion complications, please keep this definition in mind.

We are interested in abortion complications that occur to women residing in Zimbabwe, whether they obtained the abortion in or outside of the country.

301	Which medical complications resulting from induced abortion are common in Zimbabwe?<br><br>**[Interviewer: Please circle all that apply. Do not prompt.]**	A	Incomplete abortion
		B	Excessive loss of blood
		C	Anemia
		D	Infection of uterus and/or surrounding areas (sepsis)
		E	Septic shock
		F	Injury to the vagina/cervix
		G	Injury to the uterus (i.e. perforation)
		H	Injury to the internal organs
		I	Hysterectomy
		J	Infertility
		K	Death
		X	Other *(specify)* :________________________<br>____

The next set of questions use the same four subgroups of women we talked about before: rural and urban, and poor and non-poor.

COMPLICATIONS

302-305

I want to know about how likely complications are among women across each of the three methods of abortion we discussed earlier (surgical, misoprostol and all other types) and with each type of provider.

Think first about women who have surgical abortions.

Among [SUBGROUP] women who have an surgical abortion by [PROVIDER], what percent would experience a complication that needs medical treatment in a facility, **regardless of whether or not they actually receive treatment?**

[Interviewer: Ask for each type of provider and subgroup of women separately; insert the respondent's answer, even though it might be "00." If respondent says they do not know, draw a line through the boxes.

	Women who have surgical abortions	
	**a. Doctor**	**b. Nurse/midwife or other trained provider (not a doctor)**
302. Poor urban women	☐☐☐	☐☐☐
303. Non-poor urban women.	☐☐☐	☐☐☐
304. Poor rural women	☐☐☐	☐☐☐
305. Non-poor rural women	☐☐☐	☐☐☐

306-309

[If respondent answered 0% to question Q213b, Q214b, Q221b, Q222b, do not ask about misoprostol for that subgroup.]

Now I want you to think about **women who obtain abortions using misoprostol.**

Among [SUBGROUP] who have an induced abortion using misoprostol by [PROVIDER], what percent would experience a complication that needs medical treatment in a facility**, regardless of whether or not they actually receive treatment**?

[Interviewer: Ask for each type of provider and type of method separately; insert the respondent's answer, even though it might be "00." If respondent says they do not know, draw a line through the boxes.

	Women who have abortions using misoprostol abortions				
	**a. Doctor**	**b. Nurse/midwife or other trained provider (not a doctor)**	**c. Traditional provider (anyone without formal training)**	**d. Pharmacist**	**e. Woman herself - self-induced**
306. Poor urban women					
307. Non-poor urban women					
308. Poor rural women					
309. Non-poor rural women					

310-313

Now I want you to think about **women who obtain abortions using any other type of methods.**

Among [SUBGROUP OF WOMEN] who have an induced abortion using any other type of method by [PROVIDER], what percent would experience a complication that needs medical treatment in a facility, **regardless of whether or not they actually receive treatment**?

[Interviewer: Ask for each type of provider and type of method separately; insert the respondent's answer, even though it might be "00." If respondent says they do not know, draw a line through the boxes.

	Women who have any other type of abortion				
	**a. Doctor**	**b. Nurse/midwife or other trained provider (not a doctor)**	**c. Traditional provider (anyone without formal training)**	**d. Pharmacist**	**e. Woman herself - self-induced**
310. Poor urban women					
311. Non-poor urban women					
312. Poor rural women					
313. Non-poor rural women					

TREATMENT

Now I'd like you to think about whether or not women seek medical treatment in a health facility for complications from abortion.

Women may be more or less likely to seek treatment in a health facility based on what type of abortion procedure they may have had. I will ask you separately about the three types of methods (surgical, medical, and other types of abortion) for each of the four sub-groups of women we have been discussing.

314

First, think about women who had **surgical abortions**.

Among all **[SUBGROUP]** who experience a medical complication due to a surgical abortion, what percentage do you think would be treated in a health facility?

[Interviewer: Ask this question for each of the four sub-groups of women, making it clear which sub-group you are referring to and that this is only for women who had surgical abortions].

Surgical abortions

Percent of complications treated in a health facility

a. Poor urban women		%
b. Non-poor urban women		%
c. Poor rural women		%
d. Non-poor rural women		%

315	Now think about women who had abortions using **misoprosto**l.<br><br>Among all [SUBGROUP] who experience a medical complication due to an abortion using misoprostol, what percentage do you think would be treated in a health facility?<br><br>**[Interviewer: Ask this question for each of the four sub-groups of women, making it clear which sub-group you are referring to and that this is only for women who had abortions with misoprostol].**	**Misoprostol**<br>**Percent of complications treated in a health facility**<br><br>a. Poor urban women [ ][ ][ ] **%**<br>b. Non-poor urban women [ ][ ][ ] **%**<br>c. Poor rural women [ ][ ][ ] **%**<br>d. Non-poor rural women [ ][ ][ ] **%**
316	Now I'd like you to think about women who had **any other type of abortion.**<br><br>Among all [SUBGROUP] who experience a medical complication due to ay other type of method, what percentage do you think would be treated in a health facility?<br><br>**[Interviewer: Ask this question for each of the four sub-groups of women, making it clear which sub-group you are referring to and that this is only for women who had any other type of abortion].**	**Other types of abortion**<br>**Percent of complications treated in a health facility**<br><br>a. Poor urban women [ ][ ][ ] **%**<br>b. Non-poor urban women [ ][ ][ ] **%**<br>c. Poor rural women [ ][ ][ ] **%**<br>d. Non-poor rural women [ ][ ][ ] **%**

Now, I will ask you questions about all women. I will start by asking about miscarriages, also called spontaneous abortions, where a pregnancy ends by itself without anyone doing anything to end it.

317	a. Of all women who have a spontaneous abortion at **13-21 weeks** gestation, what percentage seek treatment in a health facility?<br><br>b. What about women at **6-12 weeks** gestation?	**a. Second trimester**<br>[ ][ ][ ] %<br>**b. First trimester**<br>[ ][ ][ ] %
318	The preceeding sections included questions that required you to give your opinion on concepts that are not easily measured. On a scale of 1 to 10, with 1 being "not at all sure" and 10 being "very sure", what is your degree of certainty that the answers you've given reflect the real situation encountered in your province for abortions in **urban areas**?<br><br>On a scale of 1 to 10, with 1 being "not at all sure" and 10 being "very sure", what is your degree of certainty that the answers you've given reflect the real situation encountered in your province for abortion in **rural areas**?	**a.For urban areas**: 10 Very sure, 9, 8, 7, 6, 5, 4, 3, 2, 1 Not at all sure<br><br>**b.For rural areas**: 10 Very sure, 9, 8, 7, 6, 5, 4, 3, 2, 1 Not at all sure
319	**[Interviewer: Only ask if respondent answered questions above on misoprostol use for either urban or rural areas)].**<br><br>What is your degree of certainty that the answers you've given reflect the real situation encountered in your province for **misoprostol use** in **urban areas**?<br><br>On the same scale of 1 to 10, with 1 being "not at all sure" and 10 being "very sure", what is your degree of certainty that the answers you've given reflect the real situation encountered in your province for **misoprostol use** in **rural areas**?	**a. Misoprostol use in urban areas**: 10 Very sure, 9, 8, 7, 6, 5, 4, 3, 2, 1 Not at all sure<br><br>**b. Misoprostol use in rural areas**: 10 Very sure, 9, 8, 7, 6, 5, 4, 3, 2, 1 Not at all sure

Section 4: General

Thank you again for your participation in this survey. We are nearing the end of the questionnaire and I have just a few more questions for you.

No.	Question	Code	Response
401	A lot of women fall pregnant without intending to. Please mention any suggestions or recommendations that you feel could be used in Zimbabwe to lower the level of unintended pregnancies, that is pregnancies that were unwanted or mistimed. . **[Interviewer: Do not read. Multiple responses are allowed.]**	A	Increase availability and accessibility of family planning services
		B	Improve the quality of contraceptive counseling and services
		C	Improve the provision of contraceptive counselling post delivery
		D	Improve the provision of contraceptive counselling post-abortion
		E	Implement campaigns to educate public about where to access family planning services
		F	Improve provision of education in schools, universities and communities
		G	Improve provision of reproductive health education in schools, universities, and communities
		H	Improve girls' education and women's empowerment
		I	Encourage male involvement in family planning
		J	Increasing availability of information about long-acting reversible contraceptives (such as IUDs or implant)
		K	Increase access to long-acting reversible contraceptives (such as IUDs or implant)
		X	Other (*specify*): ________
402	Please mention any suggestions/ recommendations that you feel could be used in Zimbabwe to reduce the number of unsafe abortions and their consequences for women's health. **[Interviewer: Do not read. Multiple responses are allowed.]**	A	Improve the coverage and quality of post-abortion care services
		B	Publicize the health risk involved in unsafe abortion
		C	Liberalize the legal status of abortion
		D	Further restrict the conditions under which abortion is legal
		E	Prosecute unsafe abortions
		F	Improve information and access to contraception
		G	Improve access to safe abortion services
		H	Encourage male involvement in abortion-related services
		I	Discourage premarital sex
		J	Discourage abortion (e.g. through religious teaching)
		K	Provide free reproductive health services
		L	No opinion
		X	Other (*specify*): ________
		Z	Don't know

403	Under what conditions in Zimbabwe is abortion legal?<br><br>**[Interviewer: Do not read. Multiple responses are allowed.]**	A	If the woman's physical health is at risk			
		B	If pregnancy is from rape			
		C	If pregnancy is from incest			
		D	If the foetus would be handicapped/foetal anomaly			
		E	If a girl is under age 16			
		F	Economic reasons (e.g. cannot care for the child)			
		G	If the girl or woman is unmarried			
		H	If the woman is mentally incapacitated			
		I	If the woman's mental health is at risk			
		J	If pregnancy is from contraceptive failure			
		K	If the girl or woman is still in primary or secondary school			
		L	If the woman doesn't want the pregnancy			
		M	If the woman is HIV positive			
		N	Under no circumstances			
		X	Other (specify):			
404	Do you think abortion should be allowed in the following situations?<br><br>**[Interviewer: Please read out each situation and circle respondent's answer: Yes, No, or Don't Know/DK.]**	**Situations**		No	Yes	D/K
		A	If the woman's physical health is at risk	0	1	8
		B	If pregnancy is from rape	0	1	8
		C	If pregnancy is from incest	0	1	8
		D	If the foetus would be handicapped/foetal anomaly	0	1	8
		E	If a girl is under age 16	0	1	8
		F	Economic reasons (e.g. cannot care for the child)	0	1	8
		G	If the girl or woman is unmarried	0	1	8
		H	If the woman is mentally incapacitated	0	1	8
		I	If the woman's mental health is at risk	0	1	8
		J	If pregnancy is from contraceptive failure	0	1	8
		K	If the girl or woman is still in primary or secondary school	0	1	8
		L	If the woman doesn't want the pregnancy	0	1	8
		M	If the woman is HIV positive	0	1	8
		X	Other (specify):___________________________	0	1	8

END

Thank you very much for your time. Your views are very important and will help make health services better for women.

If you are interested in receiving the final publication based on this survey after it is completed, we can give you our website address where we will be publishing the study when it is available. Would you like us to give that to you?

[Interviewer : Please fill out the section below upon completion of the interview.]

M9 **TIME FINISHED:** __ __ h __ __ m

M10 How would you classify the respondents's knowledge on the topic of induced abortion in Zimbabwe?
1= Knows very well
2= Knows moderately well
3= Has limited information
4= Doesn't know

a. National level knowledge	1	2	3	4
b. Provincial level knowledge	1	2	3	4
c. Rural level knowledge	1	2	3	4
d. Misoprostol use	1	2	3	4

Interviewer observations:

Health Professionals Survey Process Completion Checklist

Please verify that each task below has been completed. If you are responsible for the task, add your code to the corresponding task and date when the activity was completed.

	Field level				Office level	
**Questionnaire checked for:**	Interviewer	Date	Coordinator	Date	Data entry clerk signature	Date
**a. Questionnaire completeness checked**						
**b. Clear handwriting checked**						
**c. Appropriate response type checked**						
**d. Skip patterns properly followed**						

I verify that all of this information is completed.
Coordinators's signature: ______________________

FIRST DATA ENTRY COMPLETE: ______________________

SECOND DATA ENTRY COMPLETE: ______________________
