# Supplementary material for: Abortion in Zimbabwe: A national study of the incidence of induced abortion, unintended pregnancy and post-abortion care in 2016
Source: PLoS One. 2018 Oct 24;13(10):e0205239. doi: 10.1371/journal.pone.0205239 (PMC6200425; doi:10.1371/journal.pone.0205239)
Supplement: S4 File — (DOCX) [file pone.0205239.s004.docx]

**Abortion incidence, morbidity and quality of post-abortion care in Zimbabwe**

# Sub-groups of women

**Urban**

**Poor**

**Women**

**Urban**

**Non-poor Women**

**Rural**

**Non-poor Women**

**Rural**

**Poor Women**

# Types and providers of abortion

**Surgical abortions**

**Examples:** Electric or manual vacuum aspiration (MVA); Dilation and sharp curette (D&E or D&C); Catheter in a surgical setting

**Providers:**

- Doctor
- Nurse/midwife or other trained provider

**Misoprostol abortions**

**Providers:**

- Doctor
- Nurse/midwife or other trained provider
- Traditional provider (anyone without formal training)
- Pharmacists
- Women (self-induced)

**All other types of abortions**

**Examples:** Local herbs or teas; pharmaceuticals (aspirin, chloroquine etc); Sticks or metal objects inserted in the vagina etc.

**Providers:**

- Doctor
- Nurse/midwife or other trained provider
- Traditional provider (anyone without formal training)
- Pharmacists
- Women (self-induced)
