# Supplementary material for: Abortion in Zimbabwe: A national study of the incidence of induced abortion, unintended pregnancy and post-abortion care in 2016
Source: PLoS One. 2018 Oct 24;13(10):e0205239. doi: 10.1371/journal.pone.0205239 (PMC6200425; doi:10.1371/journal.pone.0205239)
Supplement: S1 Appendix — (DOCX) [file pone.0205239.s005.docx]

# **Appendix A: Mathematical appendix for the Abortion Incidence Complications Methodology (AICM)**

1. Abortion rate per 1,000 women
   1. Calculate the post-abortion care (PAC) caseloads by region: $c_{i}$ is the regional total number of women treated for abortion complications in Zimbabwe in 2016, which is calculated as the sum of the facility-level averages of PAC cases seen in facilities ($c_{ji})$ estimated in both the Health Facilities Survey (HFS) and Prospective Morbidity Survey (PMS), and supplemental Ministry of Health and Child Care (MoHCC) Monitoring & Evaluation (M&E) data.

$$c_{i}= \sum_{j} c_{ji}$$

$c_{j}$ = facility-level abortion complications derived from the average of HFS past year, HFS average year, and PMS yearly cases

$i$ = region

- 1. Remove the number of miscarriage complications, $c_{i}^{S}$: we assume that only late miscarriages (13-22 gestational weeks) would require treatment in a health facility. This requires (i) estimates of the number of second trimester miscarriages and (ii) the estimated percentage of those miscarriage complications that would receive treatment in a health facility.
     1. Calculate the number of miscarriages requiring treatment (i.e. second trimester miscarriages).

$$c_{i}^{St} =\frac{x}{100} * b_{i}$$

$c_{i}^{St}$= number of late miscarriages requiring treatment by region

$x$ = 3.41 late miscarriages per 100 live births [1]

$b_{i}$ = number of live births by region [2,3]

- - 1. We assume that only a certain proportion of late miscarriages will receive treatment and estimate the treatment seeking rate from the HPS estimated proportion of women with late miscarriages that would seek facility care (which is equivalent to the proportion of births delivered in a health facility in the DHS). Together, this proportion is assumed to equal the proportion of reproductive age women that would have access to a health facility and seek care in order to receive treatment for a late miscarriage.

$$c_{i}^{S}= c_{i}^{St}* d_{i}$$

$c_{i}^{S}$= late miscarriage complications requiring treatment that receive treatment by region

$d_{i}$ = proportion of women with second trimester miscarriages who are estimated to seek facility care in each region, weighted by the proportion of births in each province [HPS]

- - 1. Calculate the regional number of induced abortions treated in health facilities.

$$c_{i}^{A}=c_{i}- c_{i}^{S}$$

$c_{i}^{A}$= number of induced abortions receiving treatment

- 1. Calculate the regional multipliers

$$m_{i}=\frac{1}{\sum_{k} \left( \sum_{m} \left( F_{kim}* \sum_{p} \left( D_{kimp}* A_{kimp} \right) \right)* w_{ki} \right)_{i}}$$

$m_{i}=$ regional multiplier (i.e. for every one woman treated for a complication in a health facility, the number of other women who have abortions that did not result in complications or resulted in complications that did not receive treatment in a health facility)

$D_{kimp}=$ among all women in region $i$ and subgroup $k$ who have abortions, % who obtain abortion method $m$ by provider $p$ [HPS]

(Note: $\sum D_{mp}=100\%$ within each subgroup in a region)
$A_{kimp}$= % of women who experience abortion complications from each provider, by abortion method, subgroup and region [HPS]
$F_{kim}$ = % of women with complications who receive facility-based treatment, by abortion method, subgroup and region [HPS]

$w_{ki}=$ subgroup weights generated by the proportion of each subgroup within the population, by region. [2,4]

Where:

$k =$ subgroups by residence (poor/non-poor; rural/urban)

$m=$ abortion method (surgical, misoprostol, other)

$p =$ provider (doctor, nurse/midwife, traditional practitioner, pharmacist, self)

$i =$ region (Matabeleland and Bulawayo, Mashonaland and Harare, South

Eastern Region)

(Note: Region determined by location of respondent; respondents were

not asked to answer for each region)

- 1. Calculate the regional number of induced abortions

$$A_{i}= c_{i}^{A}*m_{i}$$

$A_{i}=$ regional number of induced abortions

- 1. Calculate the national number of induced abortions

$$A=\sum_{i} A_{i}$$

Pulling together steps a-d above, the full equation for estimating the number of induced abortion in Zimbabwe in 2016 is:

$$A= \sum_{i} \left( \left( \frac{1}{\sum_{k} \left( \sum_{m} \left( F_{kim}* \sum_{p} \left( D_{kimp}* A_{kimp} \right) \right)* w_{ki} \right)_{i}} \right)* \left( \sum_{j} c_{ji}- \left( x * b_{i}* d_{i} \right) \right) \right)$$

Our study deviated from the regular AICM methodology by taking into account abortions that may occur outside the country to Zimbabwean women. Steps f-h show how that adjustment was made.

- 1. Calculate the regional number of induced abortions, accounting for abortions occurring outside of Zimbabwe

$A_{i}^{SA}=A_{i}+\left( \left( s_{ip}* \frac{n_{ip}}{N_{i}} \right)* A_{i} \right)$

$s_{ip}=$ % of abortions estimated to take place in South Africa or neighboring countries, by province $p$ within region $i$ [2, HPS]

$n_{ip}=$ population of women in province $p$, by region $i$ [3]

$N_{i} =$ population of women in region $i$ [3]

$A_{i}^{SA}=$ total regional number of induced abortions, including the South Africa adjustment

- 1. Calculate the national number of induced abortions, including South Africa adjustment
  $A^{SA}=\sum_{i} A_{i}^{SA}$
  2. Calculate abortion rate per 1,000 women $\left( R \right)$, and ratio per 100 live birth $\left( T \right)$

$$R = \frac{A^{SA}}{N} *1000 T= \frac{A^{SA}}{b}*100$$

## **References**

1. Harlap S, Shiono P, Ramcahran S. A life table of spontaneous abortions and the effects of age, parity and other variables. In: Porter I, Hook E, editors. Human Embryonic and Fetal Death. New York: Academic Press; 1980. pp. 145–158.

2. Zimbabwe National Statistics Agency, ICF International. Zimbabwe Demographic and Health Survey 2015: Final Report. 2016.

3. Zimbabwe National Statistics Agency (ZIMSTAT), UNFPA. Population Projections Thematic Report. Harare: ZIMSTAT; 2015.

4. Zimbabwe National Statistics Agency (ZIMSTAT). Poverty Income Consumption and Expenditure Survey 2011/12 Report. Harare: ZIMSTAT; 2013.
