# Supplementary material for: Abortion in Zimbabwe: A national study of the incidence of induced abortion, unintended pregnancy and post-abortion care in 2016
Source: PLoS One. 2018 Oct 24;13(10):e0205239. doi: 10.1371/journal.pone.0205239 (PMC6200425; doi:10.1371/journal.pone.0205239)
Supplement: S2 Appendix — (DOCX) [file pone.0205239.s006.docx]

# **Appendix B: Removing referrals from estimated caseloads by data source**

Table B1 shows the average referral rates, which is the proportion of post-abortion care (PAC) patients in the facility who are referred to a higher level facility, by facility type, for the Health Facilities Survey (HFS) and the Prospective Morbidity Survey (PMS). The HFS referral rate was derived from the estimated number of referrals in the past month or year divided by the number of PAC patients reported in the past month or year. We also asked HFS respondents what proportion of patients are likely to make it to the higher level facility after being referred. We then adjusted the reported referral rate by the proportion likely to make it to the higher level, to create the adjusted referral rate. Primary health centers reported that, on average, over half of their PAC patients (56%) were referred to a higher level facility, followed by NGO for-profit and not-for-profit facilities (27%). There were an unweighted total of nine referrals reported over the 28-days of data collection in the PMS. The average referral rate was highest in primary health centers (15%), followed by 4% in NGO for-profit and not-for-profit facilities.

**Table B1. Referral rates subtracted from caseloads for each survey**

|  | **Data Input** | | | |
| --- | --- | --- | --- | --- |
|  | **HFS** | | | **PMS ^a^** |
|  | Reported referral rate of PAC patients in past month | Proportion likely to make it to next level facility | Adjusted referral rate |  |
| **Average referral rate by facility type** |  |  |  |  |
| Primary health centers | 64% | 90% | 56% | 15% |
| District hospitals | 5% | 98% | 5% | 1% |
| Provincial hospitals | 1% | 100% | 1% | 1% |
| Central hospitals | 0% | - | 0% | 0% |
| Private hospitals | 9% | 100% | 9% | 2% |
| NGO for profit and not for profit | 29% | 93% | 27% | 4% |
| **Overall referral rate** | **22%** | **94%** | **22%** | **1%** |
| a) Unweighted PMS caseload numbers |  |  |  |  |
