# Supplementary material for: Abortion in Zimbabwe: A national study of the incidence of induced abortion, unintended pregnancy and post-abortion care in 2016
Source: PLoS One. 2018 Oct 24;13(10):e0205239. doi: 10.1371/journal.pone.0205239 (PMC6200425; doi:10.1371/journal.pone.0205239)
Supplement: S3 Appendix — (DOCX) [file pone.0205239.s007.docx]

# **Appendix C: Estimated post-abortion care caseloads by data source**

We have four post-abortion care (PAC) caseload inputs from three different data sources. This appendix describes the four inputs and sources, shows the variation among these four sources and how that affects the average caseload number, and explains our decision to use an average of the HFS and PMS to produce the final caseload numbers.

Table C1 shows the average annual PAC caseload and the total annual PAC caseload, by facility type, for each data source we collected. The Health Facilities Survey (HFS) average year estimation was the highest overall at 24,987 total annual PAC cases. This is followed by the HFS past year estimation, at 23,370 total annual PAC cases. Past HFS surveys have shown a consistent overall pattern with a higher caseload number being reported for the average month than for the past month [1].

**Table C1: PAC caseload estimates by data source and facility type, Zimbabwe 2016**

|  | **Data source** | | | | | |
| --- | --- | --- | --- | --- | --- | --- |
|  | HFS Past Year | HFS Average Year | PMS Yearly | | PMS (95% CI) | MoHCC In Sample ^b^ |
| **Average annual PAC caseload by facility type** |  |  |  | |  |  |
| Primary health centers | 4 | 7 | 9 | | (3, 16) | 5 |
| District Hospitals | 108 | 119 | 87 | | (71, 102) | 65 |
| Provincial hospitals ^a^ | 330 | 328 | 373 | | - | 380 |
| Central hospitals ^a^ | 1,469 | 1,428 | 1,158 | | - | 1,339 |
| Private hospitals | 59 | 74 | 54 | | (45, 63) | - |
| NGO for profit and not for profit | 46 | 52 | 23 | | (21, 26) | - |
| **Total annual PAC caseload by facility type** |  |  |  | |  |  |
| Primary health centers | 241 | 397 | 558 | | (170, 945) | 257 |
| District Hospitals | 9,607 | 10,589 | 7,712 | | (6,358, 9,065) | 5,701 |
| Provincial hospitals | 2,640 | 2,627 | 2,985 | | - | 3,039 |
| Central hospitals | 7,344 | 7,140 | 5,788 | | - | 6,696 |
| Private hospitals | 1,950 | 2,439 | 1,784 | | (1,493, 2,074) | - |
| NGO for profit and not for profit | 1,589 | 1,795 | 805 | | (725, 885) | - |
| **Total annual PAC caseloads** | 23,370 | 24,987 | 19,631 | | (18,120, 21,143) | - |
| **Total annual PAC caseloads + out-of-sample public facilities (2239 total cases added^c^)** | 25,609 | 27,226 | 21,870 | | (20,358, 23,381) | **-** |
| a) No standard error for PMS PAC estimates because 100% of provincial and central hospitals were included in the sample | | | | | | |
| b) MoHCC provided M&E data for 187 facilities that were in the HFS sample (all public facilities + 20 private hospitals and 6 NGO for-profit/not-for-profits). We are only including the data for the public facilities as we have data for all of them, and private hospitals/NGO data is incomplete. These caseloads were adjusted for referrals using the referral rates estimated in the HFS (Appendix B) | | | | | | |
| c) Calculations for MoHCC cases for facilities outside of sample can be found in Table 3 | | | |  |  |  |

The PMS caseload, which is the number of women who sought PAC during the 28-day study period, was the lowest count. The prospective approach has potential for undercounting because the study period may not be an average month, some patients might not be recognized as PAC patients if they are in different wards in the facility, and women may be missed if discharged quickly and if the interviewer is not at the facility to interview them [1]. We sought to minimize these errors in fieldwork, but they were likely still present. The lower PMS caseload numbers compared to HFS caseload estimates is consistent across AICM studies that use both HFS and PMS data to find the average caseload [2].

The sampling frame for the HFS was a census of PAC-capable facilities in Zimbabwe and the PMS was a stratified random sample of this census, so we calculated 95% confidence intervals around the PMS caseload numbers. The sampling rate in central and provincial hospitals was 100% for the PMS and therefore there is no standard error for these estimates.

The Ministry of Health and Child Care (MoHCC) provided Monitoring and Evaluation (M&E) data for 187 facilities that were in the HFS sample. This included all public facilities, 20 private hospitals and 6 NGO for-profit and not-for-profit facilities. Because the MoHCC data for private and NGO facilities is incomplete, we did not include those total PAC caseloads. Comparing at the facility level, the MoHCC data are underreported compared to the other data sources, particularly in district and central hospitals, and therefore we did not include the MoHCC data in the caseload average calculation.

We also received MoHCC PAC caseload data from 825 that were not in our sample. We accounted for the underreporting with an adjustment to the MoHCC caseloads, which was calculated as the ratio of HFS past month/MoHCC reported cases in the same month. We also subtracted out referrals based on referral rates reported by the facilities. This generated a total 2,239 PAC cases from out-of-sample MoHCC facilities that we then added to each data input to reach the total annual caseloads.

## **References**

1. Fetters T. Prospective Approach to Measuring Abortion-Related Morbidity: Individual-Level Data on Postabortion Patients. In: Singh S, Remez L, Tartaglione A, editors. Methodologies for Estimating Abortion Incidence and Abortion-Related Morbidity: A Review. New York: Guttmacher Institute; 2010. pp. 135–146. Available: http://citeseerx.ist.psu.edu/viewdoc/download?doi=10.1.1.360.7567&rep=rep1&type=pdf

2. Levandowski BA, Mhango C, Kuchingale E, Lunguzi J, Katengeza H, Gebreselassie H, et al. The incidence of induced abortion in Malawi. Int Perspect Sex Reprod Health. 2013;39: 88–96. doi:10.1363/3908813
